# DNA Repair in Human Pluripotent Stem Cells Is Distinct from That in Non-Pluripotent Human Cells

**DOI:** 10.1371/journal.pone.0030541

**Published:** 2012-03-06

**Authors:** Li Z. Luo, Sailesh Gopalakrishna-Pillai, Stephanie L. Nay, Sang-Won Park, Steven E. Bates, Xianmin Zeng, Linda E. Iverson, Timothy R. O'Connor

**Affiliations:** 1 Department of Cancer Biology, Beckman Research Institute, City of Hope National Medical Center, Duarte, California, United States of America; 2 Department of Stem Cell Biology, Beckman Research Institute, City of Hope National Medical Center, Duarte, California, United States of America; 3 North Bay CIRM Shared Research Laboratory for Stem Cells and Aging, Buck Institute for Age Research, Novato, California, United States of America; University of Bristol, United Kingdom

## Abstract

The potential for human disease treatment using human pluripotent stem cells, including embryonic stem cells and induced pluripotent stem cells (iPSCs), also carries the risk of added genomic instability. Genomic instability is most often linked to DNA repair deficiencies, which indicates that screening/characterization of possible repair deficiencies in pluripotent human stem cells should be a necessary step prior to their clinical and research use. In this study, a comparison of DNA repair pathways in pluripotent cells, as compared to those in non-pluripotent cells, demonstrated that DNA repair capacities of pluripotent cell lines were more heterogeneous than those of differentiated lines examined and were generally greater. Although pluripotent cells had high DNA repair capacities for nucleotide excision repair, we show that ultraviolet radiation at low fluxes induced an apoptotic response in these cells, while differentiated cells lacked response to this stimulus, and note that pluripotent cells had a similar apoptotic response to alkylating agent damage. This sensitivity of pluripotent cells to damage is notable since viable pluripotent cells exhibit less ultraviolet light-induced DNA damage than do differentiated cells that receive the same flux. In addition, the importance of screening pluripotent cells for DNA repair defects was highlighted by an iPSC line that demonstrated a normal spectral karyotype, but showed both microsatellite instability and reduced DNA repair capacities in three out of four DNA repair pathways examined. Together, these results demonstrate a need to evaluate DNA repair capacities in pluripotent cell lines, in order to characterize their genomic stability, prior to their pre-clinical and clinical use.

## Introduction

The self-renewal and differentiation properties of human pluripotent stem cells (pluripotent cells), including both human embryonic stem cells (hESCs) and induced pluripotent stem cells (iPSCs), make them promising resources for regenerative medicine. Nevertheless, before these cells can be used therapeutically, it is critical to understand the potential risks linked to cellular maintenance and transmission of genetic information. DNA repair mechanisms are responsible for preserving genomic integrity in all cell types. However, reduced repair capacities can lead to genomic instability, which has been reported in some hESC lines [1], [2] and iPSC lines [3], [4]. Therefore, determining the DNA repair capacities for DNA repair pathways in pluripotent cells is a critical issue for pre-clinical information, as well as for understanding how pluripotent cells protect their genomes from damage.

Standard DNA repair pathways in mammalian cells include base excision repair [5], [6], nucleotide excision repair [7], [8], homologous repair, single-strand annealing, non-homologous end-joining repair, mismatch repair [9], and direct DNA repair [10]. Base excision repair corrects small DNA alterations, such as oxidized bases, uracil or alkylating agent damage. Nucleotide excision repair, on the other hand, removes mainly bulky lesions (e.g., cyclobutane pyrimidine dimers) by excision of 27–29-mer oligodeoxyribonucleotides. Nucleotide excision repair is further subdivided into global genome-nucleotide excision repair and transcription coupled-nucleotide excision repair. Homologous repair, non-homologous end-joining, and single-strand annealing are three different pathways that repair DNA double-strand breaks (DSBs) [11], [12], [13]. Error-free homologous repair requires a homologous DNA template, while non-homologous end-joining does not necessarily require homology, making it error-prone. Although single-strand annealing requires a homologous template, it is mutagenic because it anneals two extensive regions of homology that flank either side of a DSB, resulting in a deletion. Mismatch repair scans the genome for mismatched bases or single-strand loops and direct DNA repair primarily removes methylation adducts. Although some repair pathways are error-prone, for all of these mechanisms, inefficient repair can result in mutation or translocation, thus reducing the fidelity of genomic information transfer.

Despite substantial progress in the field of pluripotent stem cells, little is known about the response of pluripotent cells to mutagens or their DNA repair capacities as compared to differentiated cells. Furthermore, much of the available information concerning mutation and DNA repair has been obtained using mouse embryonic stem cells (mESCs) and not hESCs. mESCs have some prominent differences that distinguish them from their differentiated counterparts. mESCs lack a G1 checkpoint [14], [15] and more readily undergo P53-independent apoptosis than do differentiated cells [16]. Therefore, mESCs are more susceptible to apoptosis than differentiated mouse cells [17]. However, mESCs are more resistant to and more efficient at repairing oxidative damage than differentiated mouse cells [18]. With respect to mutagenesis, spontaneous mESC mutant frequencies are 100-fold lower than those of mouse embryonic fibroblasts [19] indicating that mESCs have enhanced genomic stability compared to differentiated counterparts. These data suggest that there may be differences in genomic stability and DNA repair between hESCs and differentiated human cells.

On the other hand, as compared to mESCs, hESCs have a functional CDK2-dependent G1/S checkpoint [20], [21]. However, exposure of hESCs to high energy ionizing radiation at fluxes less than 1 Gy induced apoptosis that was associated with damage responses mediated by ATM, NBS1, CHEK2, and P53, in hESCs, but not in fibroblasts [22], [23]. Additional studies also suggest that hESCs [24] and fibroblasts [17] exhibit different repair responses to ionizing radiation [25] and that hESCs use homologous repair, not non-homologous end joining, as the dominant DSB repair pathway [23], [26], [27]. Single cell gel electrophoresis (comet assay) comparing the number of DNA strand breaks following exposure to hydrogen peroxide (H_2_O_2_), ultraviolet C (UVC) radiation (254 nm), γ-radiation, or DNA cross-linking agents, showed that breaks were generally repaired faster in two hESC lines examined than in differentiated human cell lines [25]. Taken together, these results support the need to investigate further the response to DNA damage, as well as the pathway-specific DNA repair capacities of human embryonic and induced pluripotent stem cells. In particular, data are lacking on the comparison of DNA repair over a range of pathways in multiple human pluripotent stem cell lines. More importantly, minimal data exist for reprogrammed iPSCs, which could serve as better candidates for clinical use if they have similar genomic stability as hESCs and maintain their pluripotency.

In this study, DNA repair was monitored in several pluripotent and differentiated cell lines and DNA repair pathways to examine sources of genomic instability in hESCs and iPSCs. These assays encompassed nucleotide excision repair, base excision repair, non-homologous end joining, single-strand annealing, and microsatellite instability. Repair capacities from hESCs and iPSCs were compared to each other and to those of non-pluripotent cells. Evaluation upon exposure to DNA damaging agents such as UVC, dimethylsulfate (DMS) and γ-radiation indicated that pluripotent cells exhibited less damage than non-pluripotent cells, but despite lower damage levels, pluripotent cells were more prone to a type of apoptosis that could be linked to anoikis [28]. This investigation provides a basis for evaluating DNA repair capacities in pluripotent cells and emphasizes the need to evaluate the DNA repair capacity of each pluripotent cell line prior to laboratory and clinical applications.

## Materials and Methods

### Cell lines

hESC lines H9, BG01 and BG01V were obtained from WiCell, Bresagen (NovoCell) and GlobalStem, respectively. Neural stem cell line NSC09, derived from H9, was obtained from Millipore. Induced pluripotent stem cell line iPSC1, derived from human foreskin fibroblast line CRL-2097 using lentiviral vectors, was obtained from Dr. James A. Thomson (University of Wisconsin-Madison) [29]. iPSC2, derived from a human lung fibroblast line using the same retroviral introduced factors, was obtained from Dr. Jiing-Kuan Yee (Beckman Research Institute). Non-pluripotent IMR90 lung fibroblasts and CRL-2097 human foreskin fibroblasts were purchased from ATCC, GM03348E human foreskin diploid fibroblasts (HF02) were obtained from the Coriell Cell Repository and HF55 (HF01) and HF51 human neonatal foreskin fibroblasts were derived from discarded tissue provided by Arcadia Methodist Hospital from an approved protocol (City of Hope IRB# 92006). Pluripotent stem cell characterization is presented in **Figures S1** and **S2**.

### Cell culture

All cell lines were cultured as recommended. Specifically, hESCs (H9, BG01 and BG01V) and iPSCs (iPSC1 and iPSC2) were cultured in mTeSR1 (StemCell Technology) on hESC-qualified Matrigel (BD Biosciences) or on irradiated mouse embryonic fibroblasts or human fibroblasts (HFs) in conditioned hESC medium (DMEM/F12) (Cellgro, 10-092-CM4), supplemented with 20% knock-out serum replacement, 0.1 mM non-essential amino acids, 2 mM L-glutamine, 20 ng/mL fibroblast growth factor (bFGF) and 0.1 mM 2-mecaptoethanol. Medium was changed daily, and cells were either mechanically harvested, or passaged with Accutase. Rho-associated kinase (ROCK) inhibitor Y-27632 was added transiently at 10 µM to the culture medium to improve iPSC2 cell survival during passaging. Prior to exposure to DNA damaging agents or transfection, pluripotent cells were transferred to Matrigel, unless otherwise noted, to remove differentiated fibroblast feeder cells. NSC09 cells were cultured in Neurobasal Medium (Invitrogen, 21103-049) conditioned with 2 mM L-glutamine, 0.1 mM non-essential amino acids, 1X B27, Leukemia inhibitory factor (LIF, 1,000 U/mL) and bFGF (20 ng/mL). Similar to above, medium was changed daily and cells were passaged with Accutase. GM03348E cells (HF02) were cultured in Minimal Essential Medium (MEM) α with Glutamax-1 (Gibco, 32571-036) supplemented with 15% fetal bovine serum (FBS) and 0.1 mM non-essential amino acids. Medium was changed daily and cells were passaged with 0.05% Trypsin-EDTA. IMR90 lung fibroblasts were cultured in MEM containing Earle's Salts and L-glutamine (Cellgro, 10-010-CV), supplemented with 10% FBS, and passaged with 0.25% Trypsin-EDTA. HF55 (HF01) and HF51 were cultured in Dulbecco's Modified Eagle's Medium (DMEM) (Cellgro, 15-017-CV) supplemented with 10% FBS and 2 mM L-glutamine and passaged with 0.25% Trypsin-EDTA.

### Plasmids and antibodies

pCMS-end, pCMS-hom-stop, pEGFP, and pEYFP-tub were gifts from Dr. R.H. Schiestl (UCLA) [30], [31]. pRL-CMV was purchased from Promega and pM1-Luc from Roche. Antibodies were purchased from vendors as follows: Santa Cruz: rabbit anti-OCT4; Millipore: mouse anti-Oct4, goat anti-SOX2, rabbit and mouse anti-γH2AX; Abcam: rabbit anti-NANOG, mouse anti-Dnmt3b; Developmental Studies Hybridoma Bank: mouse anti-SSEA4; Cell Signaling Technology: rabbit anti-caspase 3; Kamiya: mouse anti-CPD, mouse anti-6,4 photoproducts; Sigma Aldrich: mouse anti-actin; Invitrogen: Alexa 488, 568 and 647 donkey anti-mouse, rabbit and goat IgG(H+L); and LiCor Biosciences: IR Dye800 and 680 goat anti-mouse and rabbit (secondary antibodies for dot blots and Western blots).

Single Cell Gel Electrophoresis (Comet Assay). The comet assay was performed using alkaline conditions, following the recommended protocol of the Trevigen Comet Assay Kit. Images were collected on an Olympus IX81 automated inverted fluorescence microscope and comets (sample size = 100) were quantified by measuring the %DNA in each comet tail, using CometScore software (TriTek Corp).

### DNA dot blot assay

Cells were exposed to UVC radiation (10 or 20 J/m^2^) from a germicidal lamp as previously described [32]. After treatment, cells were either harvested immediately to determine DNA damage or allowed to repair for defined periods. Genomic DNA was extracted using the DNeasy DNA extraction kit (Qiagen), following the manufacturer's instructions or by standard phenol/chloroform extraction methods, as described [33]. Concentrations of cyclobutane pyrimidine dimers (CPD) and 6,4 pyrimidine-pyrimidone adducts (6,4-PP) were determined using immunological detection with DNA South-Western dot blots [34], [35]. Residual RNA was removed by DNase-free RNase A (1 µg/mL), followed by a final extraction with phenol:chloroform:isoamyl alcohol 1∶1 and centrifugation (666× g, 5 min, room temperature [RT]). DNA in the aqueous phase was then precipitated by addition of 3 volumes of ice-cold 100% ethanol, followed by a 70% ethanol wash. Genomic DNA was air-dried and dissolved in 10 mM Tris-EDTA buffer (pH 8.0) (several h, RT or overnight, 4° C). Concentrations were determined using a NanoDrop spectrophotometer. For the DNA dot blot assay, DNA samples were prepared at 1 ng/µL in DNA denaturing solution (1.5 M NaCl, 0.5 M NaOH). A positively-charged, nylon membrane (Roche) was hydrated and fixed in a dot blot apparatus (BioRad) with a Convertible Filtration Manifold System (Life Technologies). DNA (100 ng in 100 µL) was added into three replicate wells for each sample and an equal volume of 150 mM NaCl, 50 mM Tris-HCl pH 7.6 (1× TBS) was added to all other wells not containing sample. After incubation (30 min), a vacuum was used to draw out samples and the membrane was washed (3×5 min) with 1× TBS, using a vacuum to remove each of the washes. The membrane was then air dried for 15 min, after which it was incubated with 2% blocking solution (Roche), diluted in 1× TBS (1 h, RT). The membrane was incubated with primary antibody (mouse anti-CPD or 6,4 photoproduct; 1∶2000 (Kamiya) prepared in 1% blocking solution (1 h, RT or overnight, 4° C), washed 3 times for 5 min each with 1× TBS-T (Tween-20, 1∶1000), and incubated with secondary antibody (near-IR dye 800 CW goat anti-mouse IgG; 1∶20,000) in 1% blocking solution (1 h, RT). After incubation with secondary IR-antibody, the membrane was washed again in 1× TBS-T (3 times, each for 5 min), and subjected to infrared detection by a Li-Cor Odyssey Infrared Imager. The images were quantified by TotalLab Analysis software (TotalLab Ltd.). For DNA repair assays using antibody detection, the initial ESS/Mb at time = 0 h obtained were: H9, 4.6±0.5; BG01, 6.3±0.1; iPSC1, 6.2±0.2; iPSC2, 3.2±0.2; human skin fibroblasts (CRL-2097), 25.5±1.1; human lung fibroblasts (IMR90), 14.5±0.3; and human foreskin fibroblasts (HF51), 13.9±0.4.

Live Cell Imaging. UVC-irradiated (or unirradiated) H9 cells, on Matrigel-coated chamber slides, were imaged in the live cell chamber (37° C; 5% CO_2_) of a Zeiss Axio Observer Z1 inverted microscope and live-cell imaging station. DIC images were taken at 30 min intervals. Images and movies were compiled with Image Pro 7.0.

### Annexin V apoptosis assay

Apoptosis was assessed using the Annexin V-FITC Apoptosis Detection Kit I (BD Pharmingen). H9 cells, in cold 1× PBS, were irradiated with UVC and refreshed in mTeSR1 immediately after exposure. Floating and adherent cells were collected separately at 3 and 22 h by centrifugation (500× g, 5 min) or by exposure to Accutase followed by centrifugation (500× g, 5 min), respectively. Cell pellets were washed once with and resuspended in 0.5 mL 1× PBS prior to addition of 5 µL Annexin V-FITC and 5 µL propidium iodide (PI). Control unstained, Annexin V only and PI only cells were also prepared to establish gating parameters. FACS analysis was performed on a MoFlo™ MLS cell sorter and data processed with Summit v4.3.

### DNA fragmentation to detect apoptosis

H9 or iPSC2 cells (1–2×10^6^ cells) in 35-mm culture dishes were irradiated with UVC (0 or 10 J/m^2^) in 1× PBS and incubated in fresh medium (3, 5, and 24 h, 37° C). Samples were collected as control (0 J/m^2^) or treated (10 J/m^2^) at each time point. Additionally, cells were treated with staurosporine (STS) (1 µM, 3 h, 37° C) as a positive control for apoptosis. Medium containing floating cells and attached cells was centrifuged (1000× g, 5 min) and collected as the floating fraction (F) or attached fraction (A). DNA was isolated using DNeasy Blood and Tissue kit (Qiagen), heated (10 min, 65° C), and immediately loaded onto a 1% agarose gel for electrophoresis (100 V, 2 h).

### Western blot analysis

H9 or iPSC2 cells (1–2×10^6^ cells) in 35-mm culture dishes were irradiated in 1× PBS with UVC (0 or 10 J/m^2^) and incubated in fresh medium (3, 5 and 24 h, 37° C). Samples were collected as control (0 J/m^2^) or treated (10 J/m^2^) at each time point. Additionally, cells were treated with STS (1 µM, 3 h, 37° C) as a positive control for apoptosis. The medium containing non-adherent cells was centrifuged (500× g, 10 min) to pellet floating cells. To harvest protein, 100–200 µL RIPA buffer (50 mM Tris-HCl [pH 7.4], 150 mM NaCl, 1% NP40, 0.25% Na-deoxycholate, 1 mM PMSF, protease inhibitor cocktail and phosphatase inhibitor cocktail) was added to floating cell pellets and to the remaining adherent cells, samples were incubated on ice (10 min) and centrifuged (14,000× g, 10 min, 4C°). Protein concentrations were determined using a Coomassie Blue protein assay (BioRad) [36]. Sample (50 µg) was combined with 5× SDS-PAGE loading buffer and dH_2_O, heated at 95°C for 5 min and loaded onto a 4–15% Mini Protean TGX SDS-PAGE gel (BioRad). Samples were transferred to a 0.2 µm PVDF membrane at 25 V for 3 h, using a wet electro-transfer method (0.2 M glycine, 25 mM Tris and 20% methanol). The membrane was blocked in Li-Cor Odyssey Infrared Imaging System Blocking Buffer (Li-Cor) (1 h, RT or overnight, 4° C), followed by incubation with anti-actin (1∶20,000) and anti-caspase 3 (1∶1000) primary antibodies (2 h, RT or overnight, 4°C) in blocking solution (50% [v/v] Odyssey Blocking Buffer/1× TBS). After primary antibody incubation, membranes were washed (3×5 min) in 1× TBS-T (Tris-buffered saline containing Tween-20 [1∶1000]) prior to addition of near-infrared secondary antibodies, diluted 1∶10,000, in blocking solution, as described for the primary antibody. Incubation in secondary antibody was conducted for 1 h at room temperature followed by 1× TBS-T washes (3×5 min). Detection was carried out using an Odyssey Imaging Station (Li-Cor) and band intensities were quantified with TotalLab Analysis software (TotalLab Ltd.).

### Cell transfection

Optimal transfection conditions for H9, neural stem cells, and other pluripotent cells were determined empirically by at least three different programs using the Amaxa Nucleofector Kit II (Lonza) for hESCs. Cells were harvested with Accutase, centrifuged (100× g, 10 min) and washed once with mTeSR1. Cell number was determined and cells were resuspended in 100 µL hESC Nucleofection Solution 2, mixed with 1–2 µg DNA/1×10^6^ cells and nucleofected with a set program (A-23 for H9 and iPSC1, A-13 for BG01, and B-16 for iPSC2 and BG01V). Cells were incubated in 500 µL pre-warmed RPMI 160 medium and immediately transferred to Matrigel pre-coated multi-well plates containing 1 mL mTeSR1 medium. Transfection of fibroblasts was performed using Lipofectamine 2000 according to recommendations from the manufacturer (Invitrogen).

### Microsatellite instability assay

Template DNA was prepared as described for the DNA Dot Blot Assay. The primers used in the assay are listed in Table S1 [37], [38]. PCR conditions were: 5 U/µL Taq polymerase (BioRad), 0.25 mM dNTP mix, 1 µM primers, 40 ng DNA template in 1× reaction buffer, run in 10 µL reactions for 30 cycles (94° C, 50 sec; 56° C, 50 sec; 72° C, 1 min) after denaturing at 95° C for 5 min. Product was analyzed on an ABI Prism 377 Sequencer and results were scored with GeneMapper software.

### Plasmid lesion assay

To determine the number of UVC radiation-generated lesions in plasmids, pM1-Luc plasmid (10 µg per sample) was irradiated with 200 J/m^2^ UVC and incubated (2 h, 37° C) with or without 1 µL T4 UV endonuclease (laboratory stock, 40 µg/mL). To determine levels of damage induced by reactive oxygen species photosensitization, pM1-Luc plasmid (10 µg per sample) in 10 mM sodium phosphate buffer (pH 7.4), containing 10 µM methylene blue was exposed to visible light (100 Watts, 10 cm distance, 3 min). Following exposure, plasmid was ethanol precipitated and incubated (2 h, 37° C) with 0.5 µL (2 U) formamidopyrimidine-DNA glycosylase (Fpg) (Trevigen). After incubation with T4 UV endonuclease or Fpg, samples were analyzed on 1% agarose gel. Damage sites were quantified with ImageJ software and the number of breaks (*n*) per molecule was calculated by the formula *n* = −ln *e*, *e* being the fraction of the remaining supercoiled DNA molecules [39].

### Host cell reactivation (HCR) assay for nucleotide excision repair and base excision repair DNA repair capacities

The Dual Luciferase Assay (Promega, E1910) was used to monitor DNA repair capacities for nucleotide or base excision repair. Cells were transfected (fibroblasts) or nucleofected (pluripotent and NSCs) with 2.4 µg pM1-Luc (damaged or undamaged with UVC or reactive oxygen species photosensitization as described in the previous section) and 0.24 µg pRL-CMV (internal control) per 1×10^6^ cells, and harvested after 24 h to quantify Firefly and Renilla luciferase activities. Briefly, transfected cells were washed with 1× PBS and lysed in 1× PLB buffer (passive lysis buffer supplied by Promega) (250 µL/well in 12-well plates for pluripotent cells and 6-well plates for non-pluripotent cells) on a shaking platform (20 min, RT). Triplicate samples from each lysate (20 µL per well) were transferred to individual wells of a 96-well plate, sequentially mixed with 100 µL Luciferase Assay Reagent II (LAR II) and 100 µL Stop and Glo in 96-well plates. Samples were analyzed with a Fluoroskan Ascent FL (Thermo Electron Corporation). Each assay was performed independently three times and the data combined according to the manufacturer's instructions (Promega).

### HCR assay for double-strand DNA repair capacity

Prior to the HCR assay, pCMS-end (non-homologous end-joining) and pCMS-hom-stop (single-strand annealing) plasmids were cleaved with Xho I and Apa I, or with Xho I and Sac II [30], [31], respectively. The double restriction-digested, linearized plasmids were confirmed as linear by verifying that the *Escherichia coli* transformation efficiency was less than 0.1% as compared to uncleaved plasmids. Cells were transfected (fibroblasts) or nucleofected (pluripotent cells and NSCs) with pEGFP, pEYFP, pCMS-end, pCMS-hom-stop, and double-digested pCMS-end or pCMS-hom-stop and harvested by trypsin or Accutase 24 h later. Upon harvesting, cells were stained with SYTOX red, to assess cell viability, resuspended in 0.5 mL cold 1× PBS and subjected to FACS analysis using a MoFlo™ MLS cell sorter. For each assay performed, an untransfected control and simultaneous transfection controls (pEGFP plasmid only and pEYFP-tub plasmid only) were analyzed to establish the correct gating and compensation settings. The laser settings used for GFP/YFP/Sytox Red were as follows: GFP: laser excitation wavelength 488 nm (500 mW) with an HQ500/10 emission filter, YFP: laser excitation wavelength 530 nm (50 mW) with an HQ600/30 emission filter, Sytox Red: laser excitation: 647 nm (60 mW) with an HQ680/30 emission filter. Data were analyzed using Summit v4.3 software (Dako Colorado, Inc.).

Other techniques used are described in **Materials and Methods S1**.

## Results

### DNA damage from UVC is less in pluripotent cells than in fibroblasts

**Figure 1:**
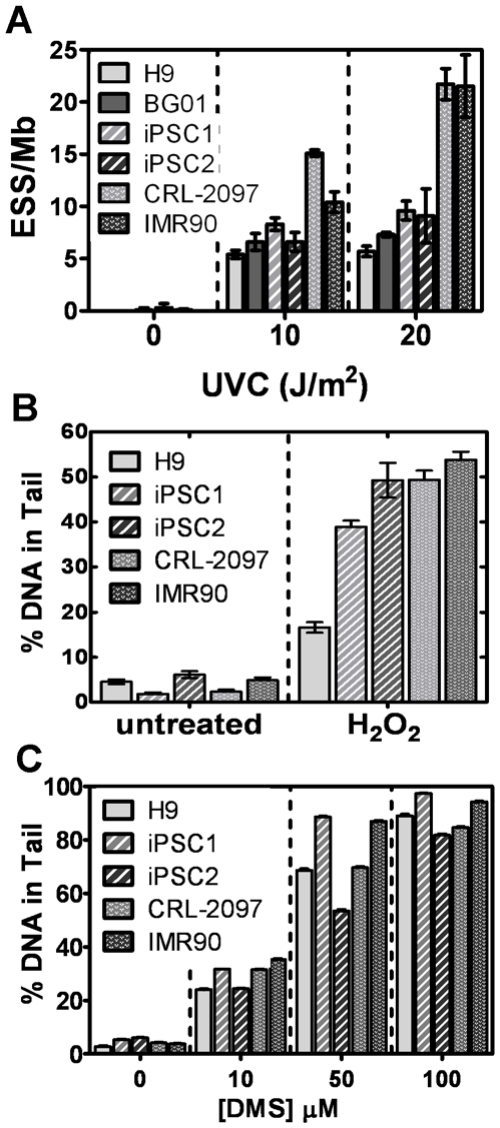
UVC-, hydrogen peroxide (H_2_O_2_)- and dimethylsulfate (DMS)-induced damage in pluripotent cells and fibroblasts. (**A**) Quantification of enzyme sensitive sites per mega base (ESS/Mb) in dot blot analysis of UVC-induced (10 or 20 J/m^2^) CPD adducts in pluripotent cells and fibroblasts. Values are mean±standard error of the mean (SEM) (n = 3). (**B**) Quantification of the percent DNA in comet tails for hESCs, iPSCs and fibroblasts treated with 100 µM H_2_O_2_. The sample size is 100 cells for each cell type and treatment; values are mean±SEM (n = 3). (**C**) Quantification of the percent of DNA in comet tails for hESCs and human skin fibroblasts treated with the indicated concentrations of DMS. The sample size is 100 cells for each cell type and treatment; values are mean±SEM (n = 3).

As a prelude to determining the DNA repair capacity for nucleotide excision repair in pluripotent cells, we examined DNA damage induced by UVC radiation (short wavelength, 100–280 nm). The levels of cyclobutane pyrimidine dimer (CPD) DNA adducts induced by UVC radiation were quantitatively measured using antibodies. H9 and BG01 ES, iPSC1 and iPSC2 induced-pluripotent, and IMR90 and CRL-2097 fibroblast cells were irradiated with UVC (10 or 20 J/m^2^), genomic DNA was isolated immediately after UVC exposure, and CPD adduct densities established (**Figures 1A** **and S4A**). CPD enzyme sensitive sites per megabase (ESS/Mb), an indication of adduct levels, were determined via alkaline gel analysis of UVC-irradiated λ DNA [40] to standardize DNA samples (**Figures S3A and S3B**). The numbers of CPD-ESS/Mb induced in pluripotent cells were 40–50% less at 10 J/m^2^ and 50–70% less at 20 J/m^2^ than those in both fibroblast lines evaluated (**Figures 1A** **and S4A**). Therefore, pluripotent cells manifest lower CPD levels than fibroblasts exposed to equal UVC fluxes.

### Reactive oxygen species-induced DNA damage is less in hESCs than in iPSCs and fibroblasts

Since UVC damage induced in pluripotent cells was less than that induced in fibroblasts, we examined the effect of treatment with other DNA damaging agents that require different pathways for repair, including hydrogen peroxide (H_2_O_2_), which causes damage that is repaired by base excision repair. Initially, hESCs, iPSCs and fibroblasts were treated using an H_2_O_2_ concentration (100 µM) that is sub-lethal to fibroblasts. Immediately after treatment, cells were harvested, lysed and analyzed by the alkaline comet assay. The relative levels of single-strand DNA breaks (SSBs), indicative of initial DNA repair were quantified as the percentage of DNA in the comet tail (%DNA Tail). Fibroblasts and iPSCs showed substantial increases in the number of SSBs (8- to 20-fold increase) after treatment as compared to untreated controls, whereas H9 cells showed only a 3-fold increase (**Figures 1B** **and S4B**). Similar to results for UVC radiation, H9 ESCs exposed to H_2_O_2_ incurred less damage than fibroblasts, but, in contrast, iPSCs had damage levels similar to those for fibroblasts.

In addition to generation of adducts repaired by the base excision repair pathway, treatment with H_2_O_2_ can lead to DSBs. Phosphorylation of Ser139 on histone H2AX is an early indicator of DSB repair that is formed at nuclear foci [41]. Therefore, to assess DSB formation as a result of H_2_O_2_ treatment in hESCs, iPSCs and fibroblasts, immunohistochemistry was used to visualize γH2AX foci formation. The number of γH2AX foci that were observed in fibroblasts was greater than in hESCs and iPSCs, indicating that fibroblasts had more strand breaks when exposed to the same amount of H_2_O_2_ damage (**Figure S5**). In contrast to results obtained with the comet assay, iPSCs showed 3- to 4-fold fewer γH2AX foci than did fibroblasts. However, iPSCs exhibited an ∼5-fold increase in γH2AX foci, compared to untreated cells and ∼2-fold more γH2AX foci than hESCs. These data are consistent with greater protection against reactive oxygen species-induced damage in pluripotent cells than in fibroblasts, with the highest protection observed in hESCs. The fold differences in γH2AX foci observed between iPSCs and fibroblasts are greater than those observed in the comet assay. This difference may be because the γH2AX foci assay generally scores DNA DSBs, whereas the alkaline comet assay monitors SSBs. These results indicate that for the cell types examined, the number of DNA strand breaks (either SSBs or DSBs) associated with base excision repair caused by H_2_O_2_ exposure was less in hESCs than in iPSCs or fibroblasts, and iPSCs had fewer or similar numbers of breaks as fibroblasts, depending on the type of break.

### Dimethyl sulfate (DMS)-induced DNA damage is variable and dependent on the pluripotent cell line

In addition to repair of reactive oxygen species-induced damage that occurs via base excision repair, we also evaluated damage generated by DMS. DMS generates principally 7-methylguanine and 3-methyladenine [42] DNA damage and these adducts also generate single-strand DNA breaks as intermediates during base excision repair. Therefore, hESCs, iPSCs and fibroblasts were incubated with DMS (0–100 µM) for 30 min and harvested immediately for alkaline comet assay analysis. When treated with 10 µM DMS, pluripotent and differentiated cells exhibited similar damage levels, quantified as %DNA Tail (**Figures 1C** **and S4C**). However, at 50 µM DMS, the %DNA Tail differed between the two iPSC lines, with iPSC1 producing larger comets than all other cell lines evaluated, including the parental line CRL-2097, whereas iPSC2 exhibited the lowest %DNA Tail. The %DNA Tail of H9 cells was lower than those of iPSC1 and IMR90, but comparable to that of CRL-2097 fibroblasts. At 100 µM DMS, the differences observed in the %DNA Tail for all the cell lines were less pronounced, but maintained a pattern similar to that at 50 µM DMS. Therefore, there were no clear differences in the damage produced by DMS in pluripotent and non-pluripotent cells. In contrast to H_2_O_2_-induced single-strand breaks, after DMS treatment, the differences in single-strand breaks observed depended on the cell line and not on whether the cells were pluripotent or differentiated.

### Global genome-nucleotide excision repair of UVC-induced CPDs is faster in pluripotent cells than in fibroblasts

**Figure 2:**
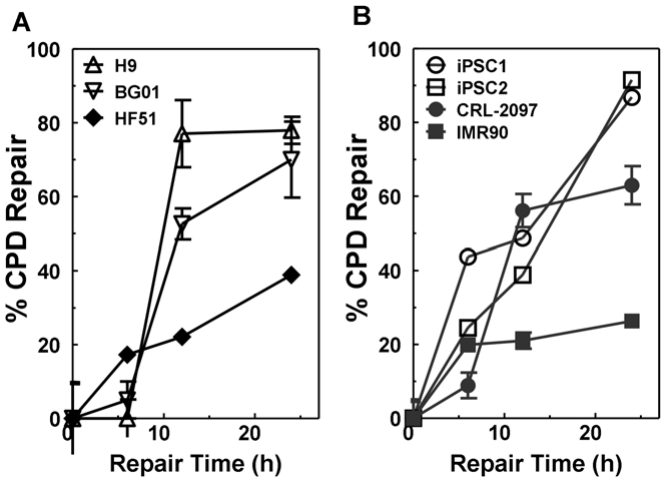
Global-genome nucleotide excision repair of UV-induced cyclobutane pyrimidine dimers (CPDs) in pluripotent cells. Quantification of global genome-nucleotide excision repair of UVC damage as percent of CPD repair in (**A**) ESCs and fibroblasts, and (**B**) iPSCs and their parental fibroblast lines. Values are mean±SEM (n = 3). The initial number of ESS/Mb following 10 J/m^2^ UVC treatment in each cell line were: H9, 4.6±0.5; BG01, 6.3±0.1; iPSC1, 6.2±0.2; iPSC2, 3.2±0.2; human skin fibroblasts (CRL-2097), 25.5±1.1; human lung fibroblasts (IMR90), 14.5±0.3; and human foreskin fibroblasts (HF51), 13.9±0.4.

Most CPD damage (∼70%) in humans is repaired by global genome-nucleotide excision repair [7]. To monitor global genome-nucleotide excision repair, we exposed pluripotent cells (H9, BG01, iPSC1 and iPSC2) and fibroblasts (IMR90, CRL-2097 and HF51) to 10 J/m^2^ UVC radiation, collected adherent cells at 0, 6, 12, and 24 h post-treatment, and isolated genomic DNA for immunoblot analysis (**Figures 2** **and S6**). We observed that over 90% of adherent cells maintained intact cell membranes, as determined by Trypan blue exclusion (data not shown). Despite the presence of fewer CPD-ESS/Mb in pluripotent cells than in fibroblasts immediately after irradiation, the DNA repair rate in pluripotent cells was greater. Specifically, H9 and BG01 hESCs were almost two times faster at repair (**Figure 2A**), and iPSC1 and 2 three times faster, than were fibroblasts (**Figure 2B**). Interestingly, for hESCs, less than 10% of CPD repair had occurred within 6 h after irradiation, with most repair occurring between 6 and 12 h. This contrasts with the rate of repair in iPSCs, which had repaired 20% of CPDs by 6 h, but had a linear type response over the 24 h period examined. This difference in the CPD repair kinetics could indicate differences in the mechanism of global genome-nucleotide excision repair between hESCs and iPSCs. We also monitored repair of 6,4 pyrimidine-pyrimidone photoproducts (6,4 PP), another UVC-induced DNA adduct. Repair of 6,4 PP was rapid for fibroblasts and pluripotent cells, with all of the adducts removed in under 2 h (data not shown). Therefore, global genome-nucleotide excision repair of CPDs induced by UVC damage was significantly greater in pluripotent cells than in fibroblasts, whereas no difference among the cell lines was observed for 6,4 PP repair rates.

### Transcription coupled-nucleotide excision repair of UVC-induced damage is faster in pluripotent cells than in non-pluripotent cells

**Figure 3:**
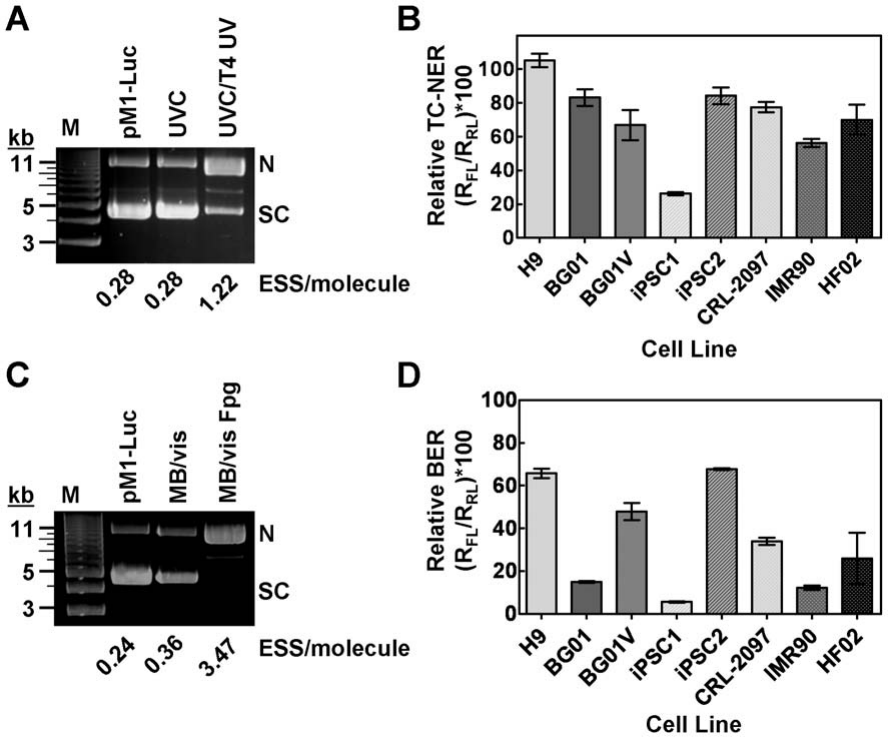
Transcription-coupled nucleotide and base excision repair in pluripotent cells determined using host cell reactivation. (**A**) Determination of the number of ESS/pM1-Luc plasmid induced by 200 J/m^2^ UVC (see Materials and Methods for details). The weak band seen between supercoiled (SC) and nicked (N) DNA is the linear form. (**B**) Host cell reactivation assay for CPD repair. Unirradiated or UVC irradiated (200 J/m^2^) pM1-Luc plasmid was co-transfected with untreated pRL-CMV plasmid (ratio of pM1-Luc/pRL-CMV was 2.4 µg/0.24 µg in 1×10^6^ cells). Dual firefly and Renilla luciferase activities were performed at 24 h post-transfection. The relative luciferase activities were compared to undamaged pRL-CMV activities. Values are mean±standard deviation (SD) (n = 3). (**C**) Determination of the number of ESS/pM1-Luc plasmid induced by methylene blue and visible light treatment (see Material and Methods for details). (**D**) Host cell reactivation assay for 8-oxo-G repair. pM1-Luc treated with methylene blue/visible light was co-transfected with undamaged pRL-CMV using the same conditions as described in (**B**). Cells were isolated 24 h post-transfection and the firefly and Renilla luciferase activities determined. Values are mean±SD (n = 3). TC-NER, transcription-coupled nucleotide excision repair; BER, base excision repair.

Since pluripotent cells exhibit low DNA damage in response to direct UVC treatment, we used host cell reactivation assays to evaluate transcription coupled-nucleotide excision DNA repair capacity in H9, BG01, BG01V, iPSC1, iPSC2, CRL-2097, IMR90 and HF02 cells. Firefly luciferase plasmid (pM1-Luc) was damaged with UVC radiation and levels of CPD damage were determined by cleavage of supercoiled DNA with T4 UV endonuclease (**Figure 3A**). An undamaged Renilla luciferase-expressing plasmid (pRL-CMV) was used as a control to normalize for transfection efficiency. The damaged firefly luciferase plasmid and undamaged control Renilla luciferase-expressing plasmid were co-transfected into the above-mentioned cells. At 24 h post-transfection, cells were harvested, lysates prepared, and firefly and Renilla luciferase activities determined using the cell extracts. The relative luciferase activities were compared to those obtained using undamaged pRL-CMV. The ratio of firefly and Renilla luciferase activities generated in cells co-transfected with the damaged pM1-Luc and control pRL-CMV was compared to the luciferase activities generated in cells co-transfected with the undamaged plasmids to reflect the ratio of repaired plasmid to intact plasmid (**Figure 3B**), which is related to the cellular DNA repair capacity. After transfection, the CRL-2097, IMR90 and HF02 fibroblast cell lines had relative luciferase activities just under 80, 60, and 70%, respectively, similar to that of BG01V (70%), while H9 and BG01 hESCs had relative luciferase activities between 80–100%. In contrast, iPSC1 and iPSC2 induced pluripotent cells exhibited significantly different relative luciferase activities, ∼25% and 80%, respectively. Therefore, a generalization on the UVC-transcription coupled nucleotide DNA repair capacity with respect to pluripotency is not possible. These results indicate that recovery of the firefly luciferase activity is dependent on the cell line, with BG01 and BG01V recovery slower than that for H9. Surprisingly, although both iPSC lines were derived from fibroblasts and with the same reprogramming factors, their DNA repair capacities were notably different.

### DNA repair capacity in base excision repair is cell line dependent

Similar to UVC, little damage was observed following H_2_O_2_ exposure of hESCs. Therefore, to monitor transcription coupled-base excision repair, we used a host cell reactivation assay analogous to that used for transcription coupled-nucleotide excision repair, described above, but using methylene blue and visible light to generate principally 8-oxoguanine *in vitro* [43]. Total 8-oxoguanine in the pM1-Luc plasmid used for transfection was estimated based on the DNA strand break frequencies induced using Fpg (**Figure 3C**). Twenty-four hours after DNA damage induction, H9, BG01V and iPSC2 exhibited superior base excision repair, with over 50% of relative firefly luciferase activity recovered, compared to CRL-2097, IMR90 and HF02 fibroblasts, which recovered between 15 and 40% of relative firefly luciferase activity (**Figure 3D**). Similar to transcription coupled-nucleotide excision repair activity, the iPSC1 cell line displayed the lowest repair efficiency. Surprisingly, the base excision repair capacity of hESC line BG01 was more similar to that of IMR90 and iPSC1 than that of the H9 hESC line. Therefore, simple classification of base excision repair solely on pluripotency is not possible.

### Non-homologous end joining DSB DNA repair capacities in pluripotent cells and fibroblasts are comparable

**Figure 4:**
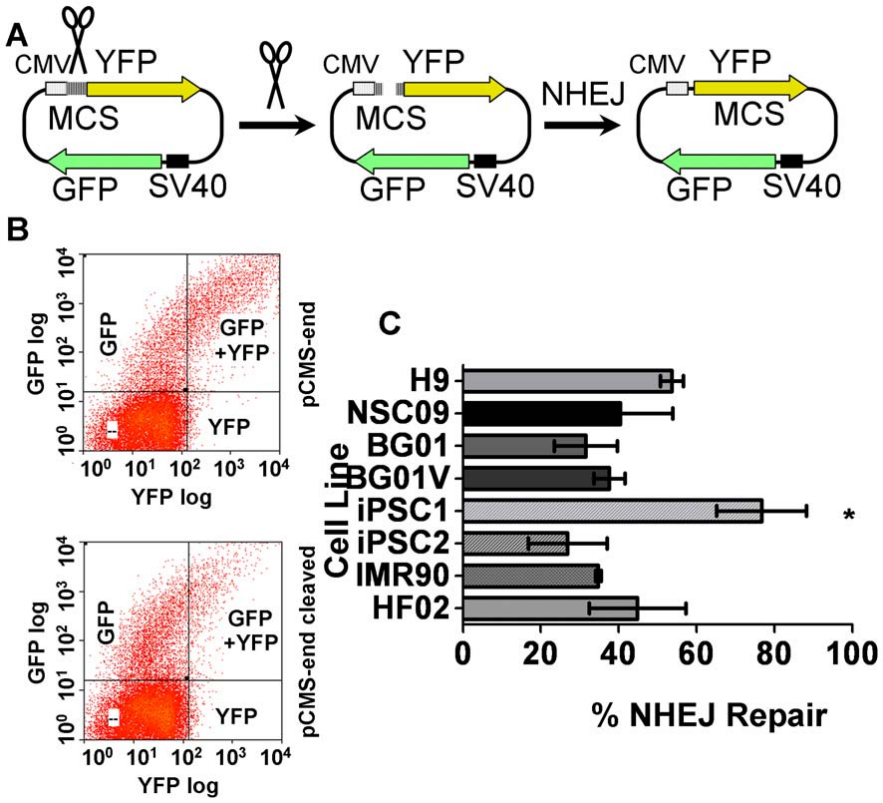
Double-strand break repair assay for non-homologous end joining using HCR. (**A**) Schematic drawing of host cell reactivation assay for non-homologous end joining repair. Non-homologous end joining is assessed by cleaving the pCMS-end plasmid (Xho I/Apa I) to generate a non-religatable DSB between the promoter and the YFP-coding region prior to transfection. Repair of the DSB by non-homologous end joining reconstitutes a link between the promoter and YFP coding region while GFP serves as an expression control. Uncleaved pCMS-end served as a positive control (100% recombination efficiency). The absolute recombination efficiency was calculated as the fraction of cells that recombined (YFP+ and GFP+YFP cells) over the total number of transfectants (YFP+,GFP+, plus GFP+YFP cells), then corrected for the “recombination efficiency.” The efficiency for non-homologous end joining was calculated as follows: ([GFP+]+[YFP+])/([GFP+]+[YFP+]+[GFP+YFP])_end-cleaved_ divided by ([GFP+]+[YFP+])/([GFP+]+[YFP+]+[GFP+YFP])_end_. (Adapted from [31]) (**B**) Representative FACS data for determination of the non-homologous end-joining DNA repair capacity in BG01. Transfected cells were harvested and assayed 24 h post-transfection. The upper panel shows FACS data for the control plasmid transfection data compared to the dual cleaved (Xho I/Apa I) plasmid in the bottom panel. (**C**) Quantification of non-homologous DSB repair capacity using host cell reactivation assays in hESCs, hESC-derived NSCs, iPSCs and fibroblasts. Values are mean±SEM (n = 3). *, statistically significant as determined by unpaired Student's t-test between iPSC1 and IMR90/iPSC2 with 2-tailed P value = 0.02 and 0.03, individually.

Non-homologous end joining is an error prone pathway for repair of DSBs. Non-homologous end joining was monitored using a transient transfection assay that did not require integration and selection (**Figure 4A**) [31]. In this analysis the GFP+YFP quadrant indicates cells that have undergone repair and produce not only the control GFP, but also the protein from the repaired YFP coding sequence. As a control, the FACS analysis of the uncleaved pCMS-end plasmid transfected into BG01 cells showed a strong GFP+YFP quadrant (**Figure 4B**, top panel). After cleavage with Apa I and Xho I, the reporter plasmid was transfected into BG01 cells, which showed significant YFP+GFP signal recovery after repair (**Figure 4B**, bottom panel). The DNA repair capacities associated with non-homologous end joining for the different cell lines showed that aside from iPSC1, the percent of non-homologous end joining repair in the cell lines investigated was less than 60% (**Figure 4C**). In contrast, iPSC1 non-homologous end joining repair was nearly 90%, a significant difference when compared to the other pluripotent and fibroblasts cells evaluated. The greater non-homologous end joining DNA repair capacity of iPSC1 also differed from the lower DNA repair capacities observed for this cell line in the nucleotide and base excision repair host cell reactivation assays. Those lower DNA repair capacities suggest that iPSC1 would manifest greater genomic instability than the other pluripotent cell lines analyzed. Taken together, the non-homologous end joining DNA repair capacities indicate that non-homologous end joining is similar among hESC and fibroblast cell lines.

### Single-strand annealing DSB repair DNA repair capacities are lower in pluripotent cells than in fibroblasts

**Figure 5:**
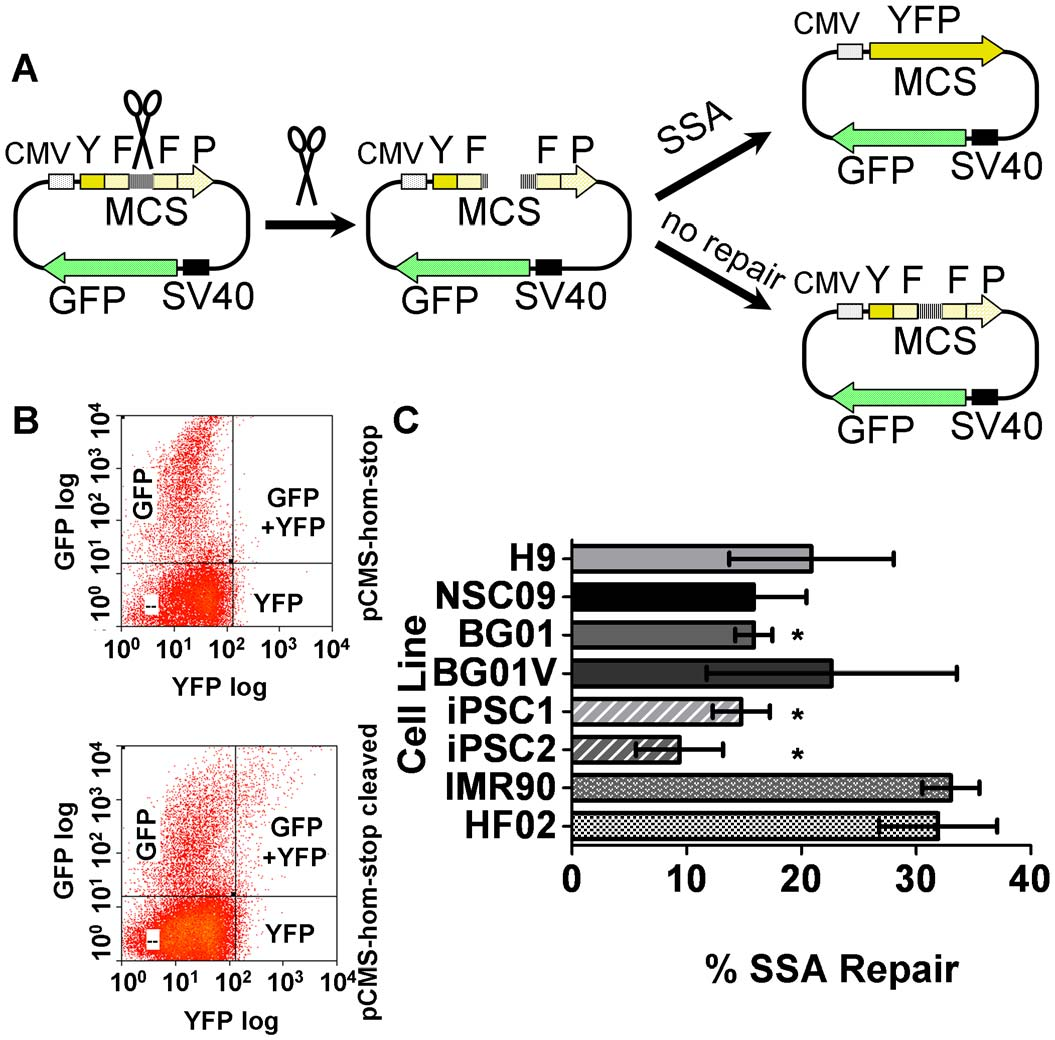
Double-strand break repair assay for single-strand annealing using host cell reactivation. (**A**) Schematic drawing of host cell reactivation assay for single-strand annealing repair. Single-strand annealing is assessed by cleaving the pCMS-hom-stop plasmid (Xho I/Sac II) to generate a DSB with incompatible ends between the 5′ and 3′ YFP coding regions prior to transfection. YFP signal was observed only when the correct reading frame was restored by single-strand annealing (adapted from [31]) (**B**) Representative FACS data for determination of single-strand annealing DNA repair capacity in BG01. The single-strand annealing efficiency is calculated as follows: ([GFP+]+[YFP+])/([GFP+]+[YFP+]+[GFP+YFP])_hom-stop_ subtracted from ([GFP+]+[YFP+])/([GFP+]+[YFP+]+[GFP+YFP])_hom-stop-cleaved_. Uncleaved pCMS-hom-stop served as zero percent recombination efficiency in the single-strand annealing assay. The absolute recombination efficiency was calculated as the fraction of cells that recombined (YFP+ and GFP+YFP+ cells) over the total number of transfectants (YFP+,GFP+, plus GFP+YFP+ cells), then corrected for the “recombination efficiency.” (**C**) Quantification of DSB repairs in hESCs, hESC-derived neural stem cells, iPSCs and fibroblasts through the single-strand annealing repair pathway by HCR assay. Values are mean±SEM (n = 3). *, statistically significant as determined by unpaired Student's t-test between IMR90 and iPSC1, iPSC2, BG01 with 2-tailed P values of 0.007, 0.006, and 0.004, respectively.

Single-strand annealing is a form of homologous recombination that involves annealing of extensive regions of homology that flank a DSB [44], which causes a deletion between the homologous segments, and hence is inherently mutagenic [45]. Using an assay similar to that described for non-homologous end joining, single-strand annealing was measured using a transfection-based assay in which the YFP coding sequence was restored by homologous regions spanning ∼300 bp on either side of the YFP-coding sequence (**Figure 5A**). Results from this assay using BG01 hESCs showed that transfection with uncleaved control plasmids generated almost no cells that co-expressed YFP and the control GFP proteins (**Figure 5B**, top panel). But a significant number of BG01 cells transfected with cleaved pCMS-hom-stop expressed YFP and GFP, representative of their single-strand annealing DNA repair capacity (**Figure 5B**, bottom panel). Comparison of the FACS analyses yielded a measurement of the single-strand annealing repair percentage (**Figure 5C**). The single-strand annealing DNA repair capacities of all pluripotent cells were consistently lower than those of fibroblasts. That DNA repair capacity was significantly lower (∼2-fold) in BG01 and both iPSC lines, suggests that single-strand annealing was not a preferred repair pathway for pluripotent cells. Therefore, the lower single-strand annealing repair capacities observed for pluripotent cells suggest that pluripotent cells develop fewer mutations due to that pathway as compared to differentiated cells.

### One iPSC line manifests microsatellite instability

**Figure 6:**
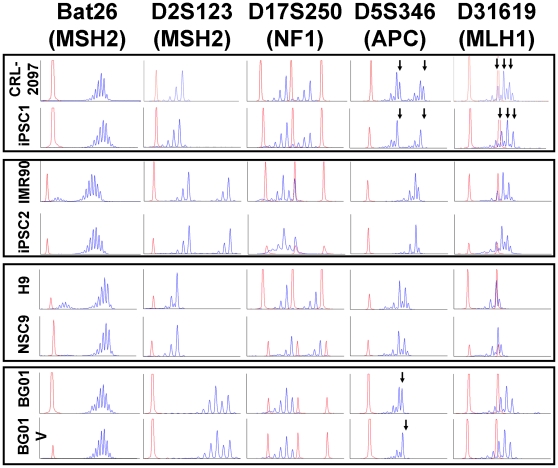
Microsatellite instability assay in pluripotent cells and differentiated cells. Comparable cell lines are grouped in black boxes, and shifts in the peaks corresponding to microsatellite instability (MSI) are marked by black arrows. The X axis shows the scan number and the Y axis shows intensity of 6-FAM. Red peaks are internal controls to indicate the locations of microsatellites. CRL-2097 are human skin fibroblasts used to generate iPSC1, IMR90 are human lung fibroblasts used to generate iPSC2, H9 are hESCs used to generate NSC9, and BG01V are an aneuploid variant hESC isolated from BG01.

Microsatellite instability (MSI) is often associated with defects in mismatch repair or DNA polymerase errors, which have been closely linked to genetic diseases that predispose individuals to cancer. Generally, identification of MSI requires comparison to reference cells that serve as an indicator of change from a starting point. Therefore, we surveyed five autosomal markers of MSI in eight cell lines, as four groups based on the relation among the cell lines (pairs consisted of CRL-2097/iPSC1, IMR90/iPSC2, H9/NSC9, and BG01/BG01V) (**Figure 6**). To evaluate differences or defects in microsatellites, the selected primer sets (**Table S1**) spanning regions near either mismatch repair genes (MSH2 [MutS Homologue 2], MLH1 [MutL Homologue 1]) or tumor suppressor genes (NF1 [neurofibromin 1], APC [adenomatous polyposis coli]) were used [37], [38]. The iPSC1 line, at passage 24, exhibited two loci with MSI, APC and hMLH1 (marked by black arrows). Additionally, BG01V, an aneuploid hESC line, had a shift in a microsatellite for the APC gene as compared to BG01 early passage cells. Overall, these MSI data show that even in iPSC1, which is karyotypically and spectral karyotypically normal (**Figure S2**), MSI is observed. Therefore, using these MSI loci is a potentially valuable tool for evaluating pluripotent cell genomic stability, as both lines that had MSI also had associated differences in either chromosomal segregation (BG01V) or in DNA repair capacities (iPSC1).

### UVC-induced apoptosis in pluripotent cells

**Figure 7:**
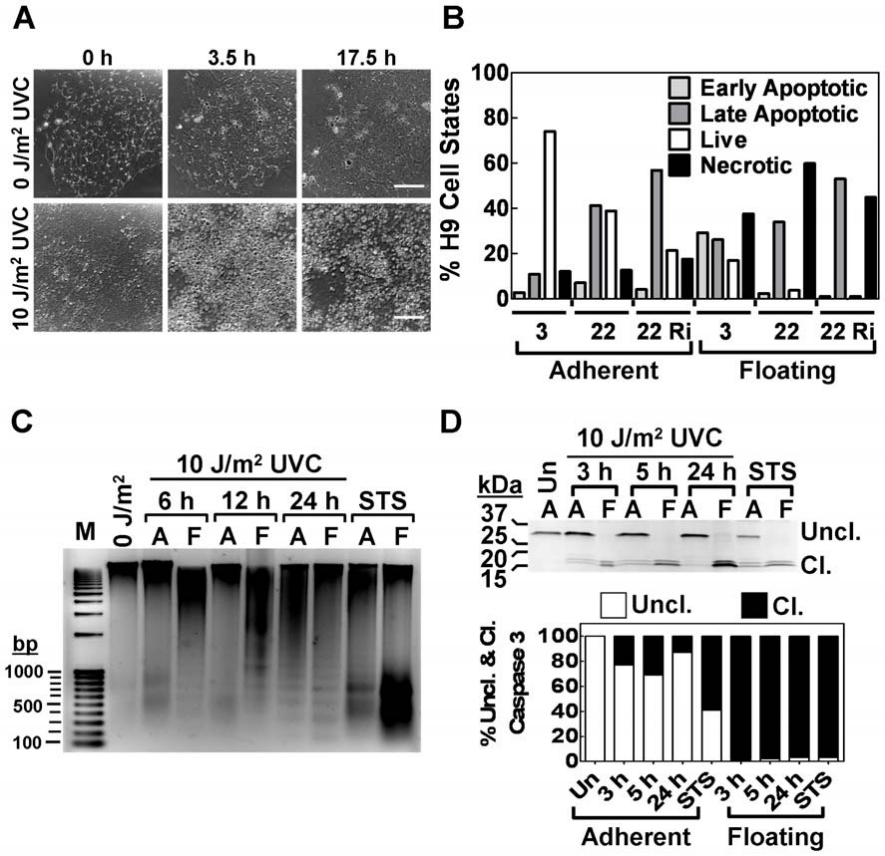
UVC-induced apoptosis in hESCs. H9 cells were treated (or not) with UVC radiation (10 J/m^2^). (**A**) Time lapse photomicrographs (differential interference contrast) of live cell cultures at 30 min intervals. Bars are 50 µm. The 3 upper panels show a control colony that was not exposed to UVC radiation. The 3 lower panels show a colony exposed to UVC radiation. The dark field in the upper panels represents the adherent attached colonies. The lower panels show that, at 3.5 h, a large number of cells from the colony are no longer adherent but float in the media. (**B**) FACS quantification of viable, Annexin V-FITC stained (early and late apoptotic) cells, and necrotic cells from either floating or adherent cells at either 3 or 22 h post-irradiation. The effect of ROCK inhibitor (Ri; 10 µM) on apoptosis was evaluated at 22 h in both adherent and floating cells. There were few if any floating cells prior to UVC exposure. Both floating and adherent cells were collected at indicated time points, labeled with the apoptosis markers Annexin V-FITC and propidium iodide and subjected to FACS analysis. Representative FACS data is in Figure S7, along with gating to indicate the different quadrants as live, early apoptotic, late apoptotic, and necrotic. (**C**) DNA fragmentation analysis of UVC-irradiated H9 cells. M, DNA markers; STS, staurosporine; F, floating cells; A, adherent cells. (**D**) Caspase 3 cleavage in adherent and floating cells. Upper panel: Western blot of caspase 3 cleavage in H9 cells treated with 10 J/m^2^ UVC (3, 5, and 24 h) or staurosporine (3 h. uncleaved (Uncl.); cleaved (Cl.); floating cells (F); adherent cells (A). Note that there were no floating cells prior to treatment. Lower panel: analysis of Western blots comparing uncleaved procaspase 3 (Uncl.) and cleaved (Cl.) bands for caspase 3.

During pilot experiments for UVC radiation exposure, we noted that pluripotent cells (hESCs and iPSCs) were more sensitive than fibroblasts, and that by 24 h post-treatment many pluripotent cell colonies had disappeared from the culture, whereas the fibroblasts underwent arrested replication [46]. In contrast to pluripotent cells, the arrest of fibroblasts was not accompanied by changes in cell death or morphology. Interestingly, the apparent pluripotent cell death following UVC exposure was characterized by detachment of cells from Matrigel, suggesting that UVC irradiation disrupted cell-cell or cell- extra cellular matrix interactions and that UVC fluxes of 10 J/m^2^ were lethal for pluripotent cells, whereas fibroblasts could recover following arrest after the same dose of UVC [47]. To further examine this phenomenon, we observed 10 J/m^2^ UVC-treated and untreated colonies over 18 h using time-lapse microscopy. H9 cells that were not irradiated with UVC proliferated and expanded, whereas UVC-irradiated colonies showed increasing numbers of detached, non-viable cells starting 3.5 h post-treatment (**Figure 7A**). Time lapse movies of this process revealed floating cells after UVC treatment (H9 hESCs) that were not observed in the controls (**Movies S1 and S2**).

To study this observation in more depth and determine cell fate after release of the H9 cells (80% confluent) from the colonies, cells were exposed to 10 J/m^2^ UVC radiation and harvested at 3 and 22 h post-treatment. Having reasoned that cell death was possibly associated with cell surface death receptors [48], we used FACS analysis to examine detached and adherent cells stained with Annexin V-FITC and propidium iodide (PI). Annexin V-FITC detects cell surface phosphatidylserines and indicates early apoptosis, whereas PI detects DNA (**Figure 7B**). The untreated H9 cells had minimal dead cells and therefore were omitted from the analysis (<2%). At 3 h post-treatment, 17% of non-adherent cells were still viable and had intact membranes, whereas 29.2% cells were entering early apoptosis. However, from 3–22 h post-treatment, the percentage of Annexin V-FITC-stained H9 cells increased in both floating and adherent cells, indicative of apoptosis. We also investigated the effect of ROCK inhibitor (10 µM) on the apoptotic response. ROCK inhibitor enhances the survival of hPSCs by improving cell-cell and cell-extra cellular matrix interactions [49], [50]. At 22 h post-treatment with the ROCK inhibitor, treated and untreated H9 cells were compared, and the adherent cells did not show significant differences in apoptosis between the two groups (**Figure 7B**). Similarly, there was no difference in the number of floating cells, regardless of whether cells were treated with the ROCK inhibitor.

To confirm that pluripotent cells were undergoing apoptosis, H9 and iPSC2 cells were exposed to 10 J/m^2^ UVC and genomic DNA was isolated separately from floating (F) and adherent (A) cells at 6, 12 and 24 h post-treatment. Genomic DNA was examined by agarose gel electrophoresis, and compared to an untreated negative control and a staurosporine-treated (1 µM, 3 h) positive control (**Figures 7C** **and S8A**). At all time points, floating cells, and adherent cells to a lesser extent, exhibited DNA ladders, indicative of apoptosis caused by endonuclease cleavage of genomic DNA.

The activated caspase 3 form is created by cleavage of procaspase 3 into 12 kDa and 17 kDa forms. The production of the cleaved procaspase 3 forms has been associated with anoikis. Anoikis is a form of apoptosis that anchorage dependent cells undergo when those detach from the extracellular matrix [51] and has been observed in hESCs [28]. Therefore, to examine further the apoptotic pathway involved in this process, we investigated procaspase 3 cleavage using Western blot analysis of protein extracts derived from either adherent or floating cells at 3, 5, and 24 post-UVC treatment (**Figures 7D** **and S8B**). At all three time points, the procaspase 3 in floating cells was completely cleaved into the active 12 kDa and 17 kDa forms. For the adherent cells, background cleavage of procaspase was noted at 3 and 5 h post-UVC treatment. However, in contrast to the floating cells, even at 24 h post-UVC treatment, procaspase 3 still formed a significant percentage of the total procaspase+caspase 3 in the adherent cells (**Figure 7D**), indicating that the remaining adherent cells were viable.

We also examined procaspase 3 cleavage in iPSC2 cells. As anticipated, the non-adherent cells showed a high percentage of caspase 3 cleavage (**Figure S8B**). However, surprisingly, a high percentage of iPSC2 cells manifested caspase 3 cleavage products at all time points in adherent cells, suggesting that the apoptotic response in iPSC2 cells was more sensitive to UVC-induced apoptosis than that of hESCs.

In addition to evaluating apoptotic response after UVC damage, we also noted that cells exposed to 50 and 100 µM DMS also detached underwent cell death associated with apoptosis (data not shown). Furthermore, such changes observed in hESCs and iPSCs exposed to γ-radiation as low as 1 Gy were recently reported [22]. In contrast to the apoptotic response caused by UVC, DMS, and γ-radiation, there was no indication of apoptosis upon H_2_O_2_ exposure in either pluripotent cells or fibroblasts. Thus, the low tolerance for DNA damage that triggers apoptosis is not observed for all damaging agents.

## Discussion

**Figure 8:**
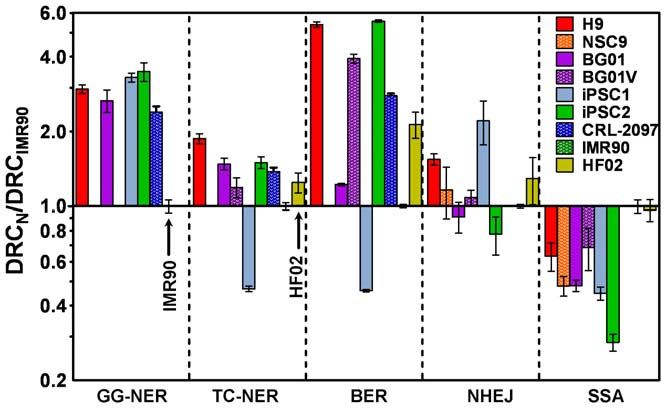
Summary of DNA repair rates/capacity in multiple DNA repair pathways in all cell lines investigated. Y axis shows the logarithmic phase of fold difference of pluripotent cells over IMR90 fibroblasts. Dotted lines are used to separate the repair pathways and direct comparisons should be limited to within the pathways. Values are mean±SD. DNA repair capacities were evaluated at 24 h after treatment or transfection.

We have shown that the DNA repair capacities of hESCs and iPSCs are greater for nucleotide excision repair and base excision repair, than are those of non-pluripotent cells. However, when evaluating DSB repair, the DNA repair capacities of non-homologous end-joining in pluripotent cells were statistically indistinguishable from those for non-pluripotent cells, except for one iPSC line. In contrast, the DNA repair capacity for single-strand annealing, which is inherently mutagenic, was lower for all pluripotent cell lines and highest in the fibroblast lines. Moreover, induction of DNA-damage in pluripotent cells by UVC and H_2_O_2_ was lower than in fibroblasts. However, in pluripotent cells, despite the reduced level of DNA damage and the rapid repair kinetics in the global genome-nucleotide excision repair pathway, exposure to UVC and DMS initiated apoptotic cell death, resulting in cell detachment at doses that are non-lethal to fibroblasts. The summarized data, comparing only data for each cell line for each assay (**Figure 8**), demonstrate the complexity of studying DNA repair in different pluripotent cell lines, and the need for characterization of these lines prior to experimental use.

### DNA damage induced by UVC and H_2_O_2_ is lower in pluripotent cells than in differentiated cells

Using either adduct detection by lesion specific antibodies or the comet assay, we showed that UVC and H_2_O_2_ induce less DNA damage in hESC lines as compared to differentiated fibroblasts (**Figure 8**). iPSC lines, however, only had less damage after UVC irradiation, and otherwise exhibited similar levels of damage to those of differentiated fibroblasts. Despite the differences in DNA damage caused by UVC and H_2_O_2_, little difference was noted for DMS treatment. The reasons for the reduced damage levels in hESCs are unclear at this time. One possible explanation is the colony structure of pluripotent cells may shield some cells from exposure to DNA damaging agents. The consequences of oxidative and methylating agent exposure in pluripotent cells also require further investigation, because, in addition to possible exposure differences, apoptosis and repair could also depend on colony architecture. Because repair metabolism causes DNA damage due to the development of reactive oxygen species and methylation [52], [53], [54], [55], future characterization of the factors that reduce DNA damage in pluripotent cells is an area that merits further examination.

### Global genome-nucleotide excision repair capacity, but not transcription-coupled nucleotide excision repair, is enhanced in pluripotent cells as compared to fibroblasts

A previous study evaluating strand breaks, as assessed by comet assay, showed that repair was faster in pluripotent than in non-pluripotent cells [25], but that study did not quantify adduct levels or viability after mutagen treatment. In this report, we have quantified UVC adduct formation and repair, and showed that the difference in the global genome-nucleotide excision repair rate is linked mainly to faster CPD adduct repair in pluripotent cells, because 6,4 PP was rapidly repaired in both pluripotent cells and the control lines. In pluripotent cells, global genome-nucleotide excision CPD repair rates were 2- to 4-fold higher than those in fibroblasts (**Figure 8**). In fibroblasts, transcription-coupled nucleotide excision repair rates are faster than global genome-nucleotide excision repair rates [56], [57], [58]. However, the transcription coupled-nucleotide excision DNA repair capacity in pluripotent cells did not exceed a 2-fold difference. Consequently, because the increased global genome-nucleotide excision DNA repair capacity is greater in pluripotent cells, the mutation frequency in pluripotent cells should be lower compared to that observed in fibroblasts. Because global genome-nucleotide excision repair rates are increased relative to transcription-coupled nucleotide excision repair rates in pluripotent cells, we anticipate that factors specific for global genome-nucleotide excision repair, possibly XPC-HR23 recognition, are responsible for faster repair rates.

### Base excision repair is faster in pluripotent cells than in non-pluripotent cells

The base excision repair rates of the pluripotent cell lines investigated demonstrated greater heterogeneity than did their transcription-coupled nucleotide excision repair rates (**Figure 8** and **Table S2**). Specifically, the lowest DNA repair rate was 13-fold less than the highest. Whereas H9, BG01V, and iPSC2 had base excision DNA repair capacities that were greater than those of the differentiated cells, BG01 and iPSC1 had lower DNA repair capacities than the differentiated cells. Moreover, the aneuploid line BG01V manifested a base excision repair DNA repair capacity that was 3-fold higher than that of the parental BG01 line. For both transcription-coupled nucleotide excision repair and base excision repair, the DNA repair capacity values were lowest in the iPSC1 line derived from CRL-2097, which indicates this line is more subject to genomic instability. The variability in the base excision repair capacities of the different lines was not separable into predictable categories of pluripotent and differentiated cells. Thus, that underscores the necessity to evaluate DNA repair capacity for each cell line prior to use in research or clinical settings.

### DSB repair is a source of differences between hESCs and iPSCs

Some studies have used stably-transfected DSB reporter assays in pluripotent cells to assess DSB repair [26], [27]. This type of system has the advantage of monitoring chromosomal events, but requires the generation of stable transfectants that host the reporter assay. At this time, introduction and selection of the stably-transfected reporter assay systems is still non-trivial in pluripotent cells and has been used for only the aneuploid BG01V line [26]. The advantage of the host cell reactivation systems is that less time is required for the assay, permitting a rapid comparison of a larger number of cell lines with respect to non-homologous end-joining and single-strand annealing. Currently, it is unclear if the source tissue of the fibroblasts (foreskin and lung) or the vectors used in re-programming account for the substantially different DNA repair capacities observed in the two iPSC lines. This highlights the importance of standardizing reprogramming protocols and minimizing additional variables that could contribute to differences exhibited during characterization.

In contrast to the results for transcription-coupled nucleotide excision repair and base excision repair, all cell lines examined, except for iPSC1, showed similar DNA repair capacities for non-homologous end-joining. Interestingly, despite having the lowest DNA repair capacity for transcription coupled-nucleotide and base excision repair, iPSC1 displayed the highest non-homologous end-joining DNA repair capacity. More surprisingly, although both iPSC1 and iPSC2 were obtained from human fibroblasts (foreskin and lung, respectively) using the same transcription factors to induce pluripotency, the non-homologous end-joining DNA repair capacity of iPSC2 is ∼3-fold less than that of iPSC1. Therefore, although the same transcription factors were used to induce both iPSC lines, each cell line had significantly different non-homologous end-joining repair characteristics. The lower values for single-strand annealing in all pluripotent cells could be associated with those cells having reduced the mutagenic consequences from repair using that pathway.

### Two pluripotent cell lines with microsatellite instability also manifest DNA repair capacity differences from other pluripotent cells

MSI is generally considered a marker for mismatch repair defects and/or DNA synthesis, which can lead to DNA mutations that are linked to human disease [59], [60], [61], [62], [63]. Since the various pluripotent lines exhibited such drastic repair capacity differences, MSI was evaluated as a candidate for these inconsistencies. Among the hESC lines, BG01V manifested MSI at a single locus. The major differences observed in BG01V are at the chromosomal level [64], but we also identified one locus adjacent to APC, a tumor suppressor gene, that differed between BG01 and BG01V. However, screening for short tandem repeat (STR) sequences did not reveal differences between these lines [65], which suggests that the MSI screening loci used in this report are more sensitive than STR analysis for genomic stability assessment. Although spectral karyotyping showed that the iPSC lines used in these experiments are normal, additional analysis of induced pluripotent lines showed that the iPSC1 line manifested greater MSI at two loci as compared to the parental line. Therefore, the differences in iPSC1 DNA repair capacity, compared to the other cell lines investigated, could be due in part to factors linked to MSI, emphasizing the need to examine pluripotent cells through other methods.

The therapeutic merits of hESCs and iPSCs are currently under evaluation and some differences among hESCs and iPSCs have been identified. Reports have indicated that iPSCs have less efficient growth and differentiation capacities than hESCs [66], distinct methylation and de-methylation patterns in non-coding RNAs [67], and variations in X-chromosome reactivation [68], suggesting that hESCs and iPSCs also have epigenetic differences. Another study showed that hESCs and iPSCs are heterogeneous, depending on their derivation source [69], and it is possible that DNA repair pathway reprogramming is dependent on the cells of origin. Our data indicate that hESCs and iPSCs have differences in DNA repair that can be monitored by a series of assays that encompass a range of DNA repair pathways. Based on our results, further work that determines the genomic stability of iPSCs is required that evaluates methods for induction of iPSCs as well as the cells of origin. The decreased repair capacities observed in the pathways studied, along with the increased MSI, suggests that iPSC1 is more prone to errors from nucleotide and base excision repair than are the other lines investigated, including iPSC2. The comparisons of MSI in the karyotypically abnormal line (BG01V) and the karyotypically normal iPSC1, indicate that screening for MSIs could provide rapid evaluation of genomic stability. The reduced DNA repair capacities manifested mainly by iPSC1, track with its MSI, suggesting that determination of MSI at these loci could also help to illuminate defects in other repair pathways.

### Pluripotent cells are subject to increased apoptosis after exposure to DNA damaging agents

Apoptosis occurs naturally in pluripotent cells grown in mTeSR1, and is enhanced by depletion of basic fibroblast growth factor [28]. Our study indicates that pluripotent cells also undergo apoptosis after low levels of exposure (e.g., 5 J/m^2^ UVC) or concentrations (e.g., 50 µM DMS) of some DNA-damaging agents. However, for H_2_O_2_, no apoptotic response was observed, indicating that not all damaging agents elicit the same programmed cell death. In addition, we showed that for up to 24 h after UVC radiation the majority of the adherent hESCs have intact membranes that lack evidence of apoptosis, but that iPSCs exhibit increased apoptotic sensitivity within 3 h post-irradiation, resulting in cells being released from colonies. To the best of our knowledge, although apoptosis has been reported in human pluripotent cells in response to γ-radiation [22], [23], [70] no previous data have addressed low-level UVC induced apoptosis in human pluripotent cells. Due to the lower energy of UVC radiation compared to γ-radiation, the observation of apoptosis was unexpected. Moreover, although ROCK inhibitor can enhance pluripotent stem cell attachment [49], its failure to rescue UVC-induced apoptosis in H9 cells suggests that the hypersensitivity to UVC-induced cell death does not involve the Rho-myosin-actin-caspase pathway, but some other apoptotic trigger, possibly CHK1 and/or CHK2 [20]. Further research is warranted to identify the mechanism in the pathways involved.

The apoptotic response of pluripotent cells to low level UVC may protect the genomic stability of the entire population by sacrificing damaged cells in a timely fashion and raises the question of evolutionary preservation in this instance, because damage tolerance in hESCs could lead to mutations if proliferation continues. Therefore, there is a paradox involved in genomic stability of pluripotent cells: DNA repair is often rapid in pluripotent cells as compared to differentiated cells, but exposure to relatively low levels of DNA-damaging agents results in cell death. In fact, the low level of UVC-induced damage tolerated by pluripotent cells shows that these cells are almost as sensitive to UVC as some human fibroblasts from individuals with defective nucleotide excision repair genes [71]. Thus, our data suggest that pluripotent cells efficiently repair damage, but will undergo cell death in the short damage response interval identified in this study rather than risk the possibility of transmitting a mutation or genetic rearrangement if the damage is not repaired. That the higher energy γ-radiation also results in apoptosis at low radiation fluxes (less than 2 Gy [22], [23], and our unpublished results]) argue against peripheral damage of colonies as a provocation of apoptosis. In the future, identification of the agents that induce apoptosis and apoptotic signaling will yield important insight concerning mechanisms of cell death in pluripotent cells.

### Conclusion

We have demonstrated that, in general, hESCs excel in global genome-nucleotide excision repair as compared to non-pluripotent cells. Using the assays described, we have shown that pluripotent cells and differentiated cells have similar repair capacities in non-homologous end joining, whereas pluripotent cells have attenuated DNA repair capacities in the single-strand annealing as compared to differentiated cells. Despite these generalities, DNA repair capacities for individual pluripotent cell lines show complexity that requires inspection of these and any other lines considered for clinical use. Furthermore, our investigation has shown that pluripotent cells are more prone to apoptosis than their differentiated progenitors, despite enhanced repair rates when exposed to UVC. This is consistent with pluripotent cells limiting mutations in their progeny. In the future, the identification of factors that enhance pluripotent cell genomic stability while limiting apoptosis will enable wider use of these cells. Most importantly, our work highlights that even though iPSCs may display a normal karyotype, microsatellite instability, which indicates general genomic instability, may predict alterations in DNA repair responses of pluripotent cells to various DNA damaging agents as compared to karyotypically normal counterparts that lack MSI. Taken together, these results identify a critical area that must be studied before the use of induced pluripotent cells can be explored further for regenerative medicine. Based on our results, requirements for pre-clinical screening for genomic instability in hESCs, and especially iPSCs, would benefit from the inclusion of assays to monitor transcription-coupled nucleotide excision repair, non-homologous end-joining, and single-strand annealing, as well as microsatellite instability.

## Supporting Information

Figure S1 **Characterization of hESCs and iPSCs.** (**A**) Immunohistochemical staining of pluripotent cell markers in hESCs and iPSCs. The indicated cell colonies were immunostained for SSEA4 (green), NANOG (red), SOX2 (purple), and DAPI (blue). Bars are 50 µm. (**B**) Immunohistochemical staining of *bona fide* pluripotent cell markers in iPSCs. iPSC colonies were immunostained for DNMT3B (green), SOX2 (red), and DAPI (blue). Bars are 30 µm. H9, iPSC1 (shown in 1A), as well as BG01 and iPSC2 (not shown) were all positively stained for the pluripotency markers (ES cell-specific transcription factors) Nanog, SOX2, and SSEA4. Both iPSC1 and iPSC2 also stained positive for DNMT3B (1B), confirming that they are *bona fide* iPSCs [1].
(TIF)

Figure S2 **Karyotypes of investigated pluripotent cell lines.** (**A**) H9 passage p 110, (**B**) BG01 p 54. BG01V (not shown) is a karyotypically abnormal (49, +12, +17 and XXY) long term cell culture variant originally isolated and characterized from BG01 cultures [2], and (**C**) iPSC2 p 11, as assessed by G-banding and (**D**) iPSC1 p 24, as assessed by spectral karyotyping (SKY) analysis. Both iPSC1 and iPSC2 were derived from human skin fibroblasts (CRL-2097) [3] or human lung fibroblasts (IMR90), respectively. The karyotypes examined for all these cells manifested 46 chromosomes in greater than 90% of the metaphase cells analyzed until at least p 110 for H9, p 54 for BG01, p 24 for iPSC1 and passage 11 for iPSC2.
(TIF)

Figure S3 **Analysis of CPD incidence in UVC-irradiated λ DNA.** UVC-irradiated Bacteriophage λ DNA was subjected to alkaline gel analysis (**A**) and quantification (**B**) of UVC-induced enzyme sensitive sites per mega base (ESS/Mb) was conducted. Hind III-digested lamda DNA are used as DNA markers.
(TIF)

Figure S4 **UVC, H_2_O_2_ or DMS-induced damage in hESC, iPSC and fibroblast cells.** (**A**) Dot blot of UVC-induced (10 or 20 J/m^2^) CPD adducts in pluripotent cells and fibroblasts, quantified in TotalLab. (**B**) Comet assays of hESCs (H9), iPSCs (iPSC1), or human skin fibroblasts (CRL-2097) treated with H_2_O_2_ (100 µM). Untreated cells were used as controls. (**C**) Comet assays of hESCs (H9), iPSCs (iPSC2), or human skin fibroblasts (IMR90) treated with DMS (50 µM).
(TIF)

Figure S5 **Evaluation of γH2AX foci formation in response to treatment with H_2_O_2_.** (**A**) Fluorescence images of hESCs (H9), iPSCs (iPSC1) and fibroblasts (CRL-2097) stained for γH2AX foci after treatment with 100 µM H_2_O_2_ (4°C for 30 min). The expanded cell shows the foci as examined in the individual cells. Controls are untreated samples. Bars are 20 µm. (**B**) Quantification of percent of cells with greater than 4 γH2AX foci.
(TIF)

Figure S6 **Dot blot assay data for global genome-nucleotide excision repair of UVC-induced cyclobutane pyrimidine dimers.** Dot blot images of CPD repair time course in (**A**) hESC (H9 and BG01), (**B**) iPSC (iPSC1 and iPSC2) and (**C**) fibroblast (CRL-2097 and IMR90) cells following 10 J/m^2^ UVC treatment. Only adherent cells were used in the assay. Quantification of enzyme sensitive sites per mega base was determined using standards loaded on each individual blot.
(TIF)

Figure S7 **FACS analysis of the H9 cell states post UVC irradiation (10 J/m^2^).** At the time points indicated, floating (F) and adherent (A) H9 cells were collected by centrifugation or accutase treatment followed by centrifugation and incubated with Annexin V-FITC and/or PI. Cells are divided by quadrants into live (FITC−, PI−), early apoptotic (FITC+, PI−), late apoptotic (FITC+, PI+) or necrotic (FITC−, PI+) sections. The quantification is shown in **Figure 7B**.
(TIF)

Figure S8 **UVC-induced apoptosis in induced pluripotent stem cells.** (**A**) DNA fragmentation analysis of UVC-irradiated iPSC2 cells. STS, staurosporine; S, supernatant; F, floating cells; A, adherent cells (**B**) Caspase 3 cleavage in adherent and floating cells. Upper panel: Western blot of caspase 3 cleavage in iPSC2 cells, treated with 10 J/m^2^ UVC (6, 12 and 24 h) or staurasporine (3 h), using near-infrared detection. Uncleaved (Uncl.); Cleaved (Cl.); Floating cells (F); Adherent cells (A). Note that there are no floating cells prior to treatment. Lower panel: analysis of Western blots comparing uncleaved (Uncl.) and cleaved (Cl.) bands for caspase 3.
(TIF)

Table S1 Microsatellite markers for MSI analysis.
(DOC)

Table S2 Summary of DNA repair rates/capacities of hPSCs and HFs in multiple DNA repair pathways investigated. The rates/capacities for all the lines are relative to the rates/capacities in IMR-90 fibroblasts (1.0). Values are mean ± Standard Deviation. Note that the repair rates are directly comparable down a column and not across rows.
(DOC)

Movie S1 hESC colony monitored over a 24 h period. Note colony growth over the period.
(AVI)

Movie S2 hESC colony exposed to 10 joules/m2 UVC and monitored over a 24 h period. Note the presence of detached (i.e., floating cells) following exposure.
(AVI)

Materials and Methods S1 (DOC)

## References

[1] Brimble SN, Zeng X, Weiler DA, Luo Y, Liu Y (2004). Karyotypic stability, genotyping, differentiation, feeder-free maintenance, and gene expression sampling in three human embryonic stem cell lines derived prior to August 9, 2001.. Stem Cells Dev.

[2] Maitra A, Arking DE, Shivapurkar N, Ikeda M, Stastny V (2005). Genomic alterations in cultured human embryonic stem cells.. Nat Genet.

[3] Martins-Taylor K, Nisler BS, Taapken SM, Compton T, Crandall L (2011). Recurrent copy number variations in human induced pluripotent stem cells.. Nat Biotechnol.

[4] Mayshar Y, Ben-David U, Lavon N, Biancotti JC, Yakir B (2010). Identification and classification of chromosomal aberrations in human induced pluripotent stem cells.. Cell Stem Cell.

[5] Memisoglu A, Samson L (2000). Base excision repair in yeast and mammals.. Mutat Res.

[6] Zharkov DO (2008). Base excision DNA repair.. Cell Mol Life Sci.

[7] Furuta T, Ueda T, Aune G, Sarasin A, Kraemer KH (2002). Transcription-coupled nucleotide excision repair as a determinant of cisplatin sensitivity of human cells.. Cancer Res.

[8] Gillet LC, Scharer OD (2006). Molecular mechanisms of mammalian global genome nucleotide excision repair.. Chem Rev.

[9] Jiricny J (2006). The multifaceted mismatch-repair system.. Nat Rev Mol Cell Biol.

[10] Eker AP, Quayle C, Chaves I, van der Horst GT (2009). DNA repair in mammalian cells: Direct DNA damage reversal: elegant solutions for nasty problems.. Cell Mol Life Sci.

[11] Moynahan ME, Jasin M (2010). Mitotic homologous recombination maintains genomic stability and suppresses tumorigenesis.. Nat Rev Mol Cell Biol.

[12] Rothkamm K, Kruger I, Thompson LH, Lobrich M (2003). Pathways of DNA double-strand break repair during the mammalian cell cycle.. Mol Cell Biol.

[13] Stark JM, Pierce AJ, Oh J, Pastink A, Jasin M (2004). Genetic steps of mammalian homologous repair with distinct mutagenic consequences.. Mol Cell Biol.

[14] Hirao A, Kong YY, Matsuoka S, Wakeham A, Ruland J (2000). DNA damage-induced activation of p53 by the checkpoint kinase Chk2.. Science.

[15] Koledova Z, Kafkova LR, Kramer A, Divoky V (2010). DNA damage-induced degradation of Cdc25A does not lead to inhibition of Cdk2 activity in mouse embryonic stem cells.. Stem Cells.

[16] Aladjem MI, Spike BT, Rodewald LW, Hope TJ, Klemm M (1998). ES cells do not activate p53-dependent stress responses and undergo p53-independent apoptosis in response to DNA damage.. Curr Biol.

[17] de Waard H, de Wit J, Gorgels TG, van den Aardweg G, Andressoo JO (2003). Cell type-specific hypersensitivity to oxidative damage in CSB and XPA mice.. DNA Repair (Amst).

[18] Saretzki G, Armstrong L, Leake A, Lako M, von Zglinicki T (2004). Stress defense in murine embryonic stem cells is superior to that of various differentiated murine cells.. Stem Cells.

[19] Cervantes RB, Stringer JR, Shao C, Tischfield JA, Stambrook PJ (2002). Embryonic stem cells and somatic cells differ in mutation frequency and type.. Proc Natl Acad Sci U S A.

[20] Barta T, Vinarsky V, Holubcova Z, Dolezalova D, Verner J (2010). Human embryonic stem cells are capable of executing G1/S checkpoint activation.. Stem Cells.

[21] Neganova I, Vilella F, Atkinson SP, Lloret M, Passos JF (2011). An important role for CDK2 in G1 to S checkpoint activation and DNA damage response in human embryonic stem cells.. Stem Cells.

[22] Momcilovic O, Choi S, Varum S, Bakkenist C, Schatten G (2009). Ionizing radiation induces ataxia telangiectasia mutated-dependent checkpoint signaling and G(2) but not G(1) cell cycle arrest in pluripotent human embryonic stem cells.. Stem Cells.

[23] Momcilovic O, Knobloch L, Fornsaglio J, Varum S, Easley C (2010). DNA damage responses in human induced pluripotent stem cells and embryonic stem cells.. PLoS One.

[24] Banuelos CA, Banath JP, Macphail SH, Zhao J, Eaves CA (2008). Mouse but not human embryonic stem cells are deficient in rejoining of ionizing radiation-induced DNA double-strand breaks.. DNA Repair (Amst).

[25] Maynard S, Swistikowa AM, Lee JW, Liu Y, Liu ST (2008). Human Embryonic Stem Cells have Enhanced Repair of Multiple Forms of DNA Damage.. Stem Cells.

[26] Adams BR, Hawkins AJ, Povirk LF, Valerie K (2010). ATM-independent, high-fidelity nonhomologous end joining predominates in human embryonic stem cells.. Aging (Albany NY).

[27] Adams BR, Golding SE, Rao RR, Valerie K (2010). Dynamic dependence on ATR and ATM for double-strand break repair in human embryonic stem cells and neural descendants.. PLoS One.

[28] Wang X, Lin G, Martins-Taylor K, Zeng H, Xu RH (2009). Inhibition of caspase-mediated anoikis is critical for basic fibroblast growth factor-sustained culture of human pluripotent stem cells.. J Biol Chem.

[29] Yu J, Vodyanik MA, Smuga-Otto K, Antosiewicz-Bourget J, Frane JL (2007). Induced pluripotent stem cell lines derived from human somatic cells.. Science.

[30] Howlett NG, Scuric Z, D'Andrea AD, Schiestl RH (2006). Impaired DNA double strand break repair in cells from Nijmegen breakage syndrome patients.. DNA Repair (Amst).

[31] Secretan MB, Scuric Z, Oshima J, Bishop AJ, Howlett NG (2004). Effect of Ku86 and DNA-PKcs deficiency on non-homologous end-joining and homologous recombination using a transient transfection assay.. Mutat Res.

[32] Bates SE, Zhou NY, Federico LE, Xia L, O'Connor TR (2005). Repair of cyclobutane pyrimidine dimers or dimethylsulfate damage in DNA is identical in normal or telomerase-immortalized human skin fibroblasts.. Nucleic Acids Res.

[33] Yarosh DB, Pena AV, Nay SL, Canning MT, Brown DA (2005). Calcineurin inhibitors decrease DNA repair and apoptosis in human keratinocytes following ultraviolet B irradiation.. J Invest Dermatol.

[34] Ford JM, Hanawalt PC (1997). Expression of wild-type p53 is required for efficient global genomic nucleotide excision repair in UV-irradiated human fibroblasts.. J Biol Chem.

[35] Yarosh DB, Boumakis S, Brown AB, Canning MT, Galvin JW (2002). Measurement of UVB-Induced DNA damage and its consequences in models of immunosuppression.. Methods.

[36] Bradford MM (1976). A rapid and sensitive method for the quantitation of microgram quantities of protein utilizing the principle of protein-dye binding.. Anal Biochem.

[37] Boland CR, Thibodeau SN, Hamilton SR, Sidransky D, Eshleman JR (1998). A National Cancer Institute Workshop on Microsatellite Instability for cancer detection and familial predisposition: development of international criteria for the determination of microsatellite instability in colorectal cancer.. Cancer Res.

[38] Nystrom-Lahti M, Sistonen P, Mecklin JP, Pylkkanen L, Aaltonen LA (1994). Close linkage to chromosome 3p and conservation of ancestral founding haplotype in hereditary nonpolyposis colorectal cancer families.. Proc Natl Acad Sci U S A.

[39] Laval J (1977). Two enzymes are required from strand incision in repair of alkylated DNA.. Nature.

[40] Zhou NY, Bates SE, Bouziane M, Stary A, Sarasin A (2003). Efficient repair of cyclobutane pyrimidine dimers at mutational hot spots is restored in complemented Xeroderma pigmentosum group C and trichothiodystrophy/xeroderma pigmentosum group D cells.. J Mol Biol.

[41] Sedelnikova OA, Rogakou EP, Panyutin IG, Bonner WM (2002). Quantitative detection of (125)IdU-induced DNA double-strand breaks with gamma-H2AX antibody.. Radiat Res.

[42] Kumar R, Hemminki K (1996). Separation of 7-methyl- and 7-(2-hydroxyethyl)-guanine adducts in human DNA samples using a combination of TLC and HPLC.. Carcinogenesis.

[43] Floyd RA, West MS, Eneff KL, Schneider JE (1989). Methylene blue plus light mediates 8-hydroxyguanine formation in DNA.. Arch Biochem Biophys.

[44] Lyndaker AM, Alani E (2009). A tale of tails: insights into the coordination of 3′ end processing during homologous recombination.. Bioessays.

[45] Kolomietz E, Meyn MS, Pandita A, Squire JA (2002). The role of Alu repeat clusters as mediators of recurrent chromosomal aberrations in tumors.. Genes Chromosomes Cancer.

[46] Latonen L, Taya Y, Laiho M (2001). UV-radiation induces dose-dependent regulation of p53 response and modulates p53-HDM2 interaction in human fibroblasts.. Oncogene.

[47] Gentile M, Latonen L, Laiho M (2003). Cell cycle arrest and apoptosis provoked by UV radiation-induced DNA damage are transcriptionally highly divergent responses.. Nucleic Acids Res.

[48] Kulms D, Schwarz T (2002). Molecular mechanisms involved in UV-induced apoptotic cell death.. Skin Pharmacol Appl Skin Physiol.

[49] Watanabe K, Ueno M, Kamiya D, Nishiyama A, Matsumura M (2007). A ROCK inhibitor permits survival of dissociated human embryonic stem cells.. Nat Biotechnol.

[50] Mollamohammadi S, Taei A, Pakzad M, Totonchi M, Seifinejad A (2009). A simple and efficient cryopreservation method for feeder-free dissociated human induced pluripotent stem cells and human embryonic stem cells.. Hum Reprod.

[51] Frisch SM, Francis H (1994). Disruption of epithelial cell-matrix interactions induces apoptosis.. J Cell Biol.

[52] Sedgwick B, Bates PA, Paik J, Jacobs SC, Lindahl T (2007). Repair of alkylated DNA: recent advances.. DNA Repair (Amst).

[53] Maynard S, Schurman SH, Harboe C, de Souza-Pinto NC, Bohr VA (2009). Base excision repair of oxidative DNA damage and association with cancer and aging.. Carcinogenesis.

[54] van Loon B, Markkanen E, Hubscher U (2010). Oxygen as a friend and enemy: How to combat the mutational potential of 8-oxo-guanine.. DNA Repair (Amst).

[55] Kryston TB, Georgiev AB, Pissis P, Georgakilas AG (2011). Role of oxidative stress and DNA damage in human carcinogenesis.. Mutat Res.

[56] Madhani HD, Bohr VA, Hanawalt PC (1986). Differential DNA repair in transcriptionally active and inactive proto-oncogenes: c-abl and c-mos.. Cell.

[57] Mellon I, Bohr VA, Smith CA, Hanawalt PC (1986). Preferential DNA repair of an active gene in human cells.. Proc Natl Acad Sci U S A.

[58] Venema J, Bartosova Z, Natarajan AT, van Zeeland AA, Mullenders LH (1992). Transcription affects the rate but not the extent of repair of cyclobutane pyrimidine dimers in the human adenosine deaminase gene.. J Biol Chem.

[59] Leach FS, Nicolaides NC, Papadopoulos N, Liu B, Jen J (1993). Mutations of a mutS homolog in hereditary nonpolyposis colorectal cancer.. Cell.

[60] Fishel R, Lescoe MK, Rao MR, Copeland NG, Jenkins NA (1993). The human mutator gene homolog MSH2 and its association with hereditary nonpolyposis colon cancer.. Cell.

[61] Masutani C, Kusumoto R, Yamada A, Dohmae N, Yokoi M (1999). The XPV (xeroderma pigmentosum variant) gene encodes human DNA polymerase eta.. Nature.

[62] Masutani C, Araki M, Yamada A, Kusumoto R, Nogimori T (1999). Xeroderma pigmentosum variant (XP-V) correcting protein from HeLa cells has a thymine dimer bypass DNA polymerase activity.. Embo J.

[63] Raha M, Wang G, Seidman MM, Glazer PM (1996). Mutagenesis by third-strand-directed psoralen adducts in repair-deficient human cells: high frequency and altered spectrum in a xeroderma pigmentosum variant.. Proc Natl Acad Sci U S A.

[64] Plaia TW, Josephson R, Liu Y, Zeng X, Ording C (2006). Characterization of a new NIH-registered variant human embryonic stem cell line, BG01V: a tool for human embryonic stem cell research.. Stem Cells.

[65] Zeng X, Chen J, Liu Y, Luo Y, Schulz TC (2004). BG01V: a variant human embryonic stem cell line which exhibits rapid growth after passaging and reliable dopaminergic differentiation.. Restor Neurol Neurosci.

[66] Feng Q, Lu SJ, Klimanskaya I, Gomes I, Kim D (2010). Hemangioblastic derivatives from human induced pluripotent stem cells exhibit limited expansion and early senescence.. Stem Cells.

[67] Chin MH, Mason MJ, Xie W, Volinia S, Singer M (2009). Induced pluripotent stem cells and embryonic stem cells are distinguished by gene expression signatures.. Cell Stem Cell.

[68] Urbach A, Bar-Nur O, Daley GQ, Benvenisty N (2010). Differential modeling of fragile X syndrome by human embryonic stem cells and induced pluripotent stem cells.. Cell Stem Cell.

[69] Utikal J, Maherali N, Kulalert W, Hochedlinger K (2009). Sox2 is dispensable for the reprogramming of melanocytes and melanoma cells into induced pluripotent stem cells.. J Cell Sci.

[70] Fan J, Wilson PF, Wong HK, Urbin SS, Thompson LH (2007). XRCC1 down-regulation in human cells leads to DNA-damaging agent hypersensitivity, elevated sister chromatid exchange, and reduced survival of BRCA2 mutant cells.. Environ Mol Mutagen.

[71] Lehmann AR, Kirk-Bell S, Arlett CF, Harcourt SA, de Weerd-Kastelein EA (1977). Repair of ultraviolet light damage in a variety of human fibroblast cell strains.. Cancer Res.

