# Correction: DNA Repair in Human Pluripotent Stem Cells Is Distinct from That in Non-Pluripotent Human Cells

**DOI:** 10.1371/annotation/9ee832bd-9ae3-4082-a16c-abf4480a541d

**Published:** 2012-05-15

**Authors:** Li Z. Luo, Sailesh Gopalakrishna-Pillai, Stephanie L. Nay, Sang-Won Park, Steven E. Bates, Xianmin Zeng, Linda E. Iverson, Timothy R. O'Connor

There was an error in the Author Contributions. LZL SLN SP XZ TRO conceived and designed the experiments.

