# Correction: DNA Repair in Human Pluripotent Stem Cells Is Distinct from That in Non-Pluripotent Human Cells

**DOI:** 10.1371/annotation/03765fa1-8075-4374-abe5-aa176c49e279

**Published:** 2012-05-15

**Authors:** Li Z. Luo, Sailesh Gopalakrishna-Pillai, Stephanie L. Nay, Sang-Won Park, Steven E. Bates, Xianmin Zeng, Linda E. Iverson, Timothy R. O'Connor

There was an error in the equal contributions designation. Li Z. Luo and Stephanie L. Nay contributed equally to this work.

