# Correction: DNA Repair in Human Pluripotent Stem Cells Is Distinct from That in Non-Pluripotent Human Cells

**DOI:** 10.1371/annotation/4c0baab8-74ef-47df-bb29-1506795e3350

**Published:** 2012-09-05

**Authors:** Li Z. Luo, Sailesh Gopalakrishna-Pillai, Stephanie L. Nay, Sang-Won Park, Steven E. Bates, Xianmin Zeng, Linda E. Iverson, Timothy R. O'Connor

There was an error in the published author list. Author Giau Hua was inadvertently excluded. The correct author byline is Li Z. Luo, Sailesh Gopalakrishna-Pillai, Stephanie L. Nay, Sang-Won Park, Steven E. Bates, Giau Hua, Xianmin Zeng, Linda E. Iverson, Timothy R. O'Connor. The author's position in the byline is sixth. The author's affiliation is 2 Department of Stem Cell Biology, Beckman Research Institute, City of Hope National Medical Center, Duarte, California, United States of America. The author's contributions are: Contributed reagents/materials/analysis tools.

The previous correction to the Author Contributions was inaccurate. The correct Author Contributions are:

Conceived and designed the experiments: LZL SLN SP LEI XZ TRO

Performed the experiments: LZL SLN SGP SEB

Analyzed the data: LZL SLN SP TRO

Contributed reagents/materials/analysis tools: SGP SP SEB GH XZ LEI TRO

Wrote the manuscript: LZL SLN SGP SEB XZ LEI TRO

Other: Contributed Equally: LZL SGP SLN

Intellectual Input: SGP 

