# Supplementary figures and images for: DNA Repair in Human Pluripotent Stem Cells Is Distinct from That in Non-Pluripotent Human Cells
Source: PLoS One. 2012 Mar 6;7(3):e30541. doi: 10.1371/journal.pone.0030541 (PMC3295811; doi:10.1371/journal.pone.0030541)

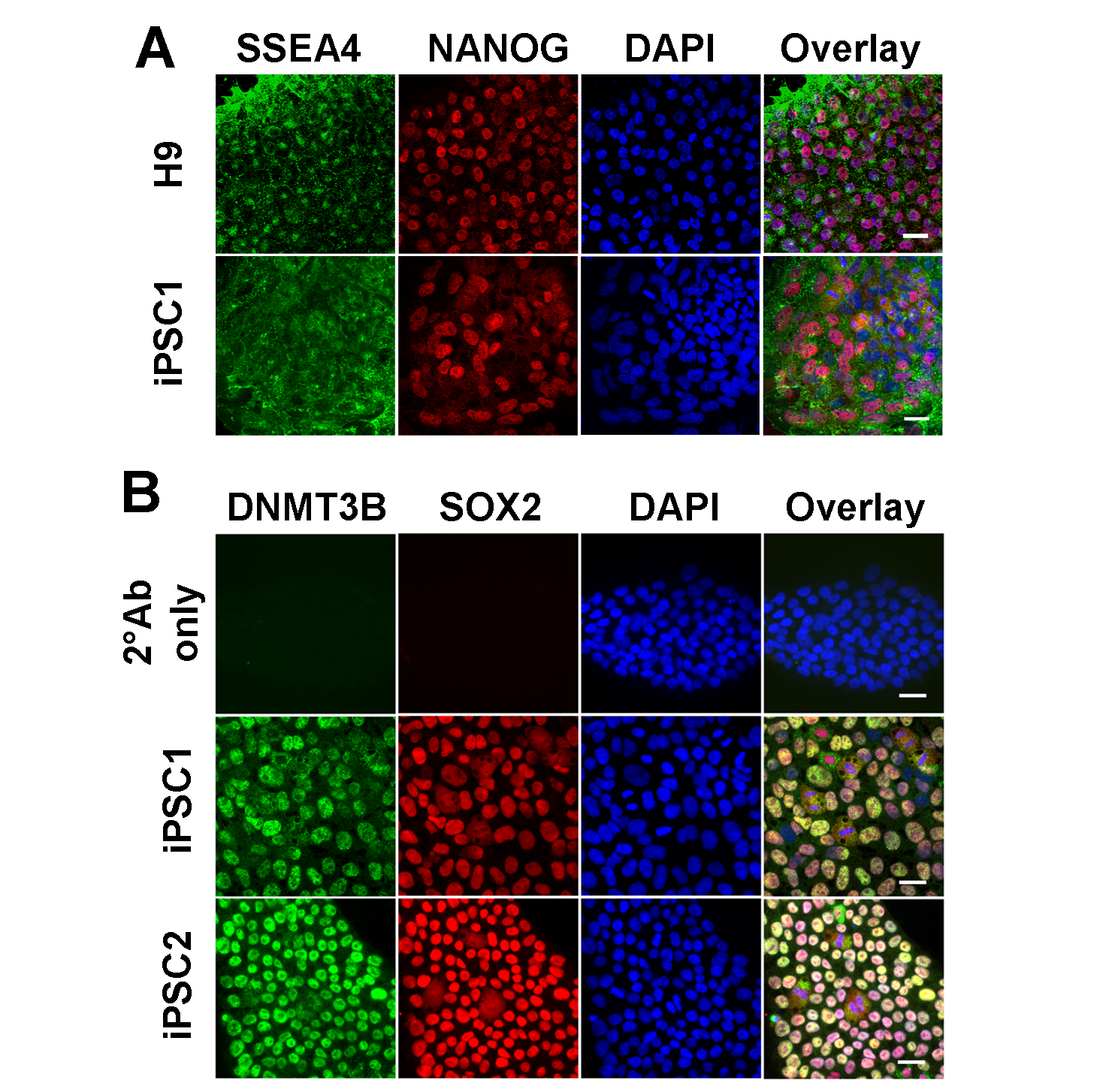

Supplement: Figure S1 — Characterization of hESCs and iPSCs. (A) Immunohistochemical staining of pluripotent cell markers in hESCs and iPSCs. The indicated cell colonies were immunostained for SSEA4 (green), NANOG (red), SOX2 (purple), and DAPI (blue). Bars are 50 µm. (B) Immunohistochemical staining of bona fide pluripotent cell markers in iPSCs. iPSC colonies were immunostained for DNMT3B (green), SOX2 (red), and DAPI (blue). Bars are 30 µm. H9, iPSC1 (shown in 1A), as well as BG01 and iPSC2 (not shown) were all positively stained for the pluripotency markers (ES cell-specific transcription factors) Nanog, SOX2, and SSEA4. Both iPSC1 and iPSC2 also stained positive for DNMT3B (1B), confirming that they are bona fide iPSCs [1]. (TIF) [file pone.0030541.s001.tif]

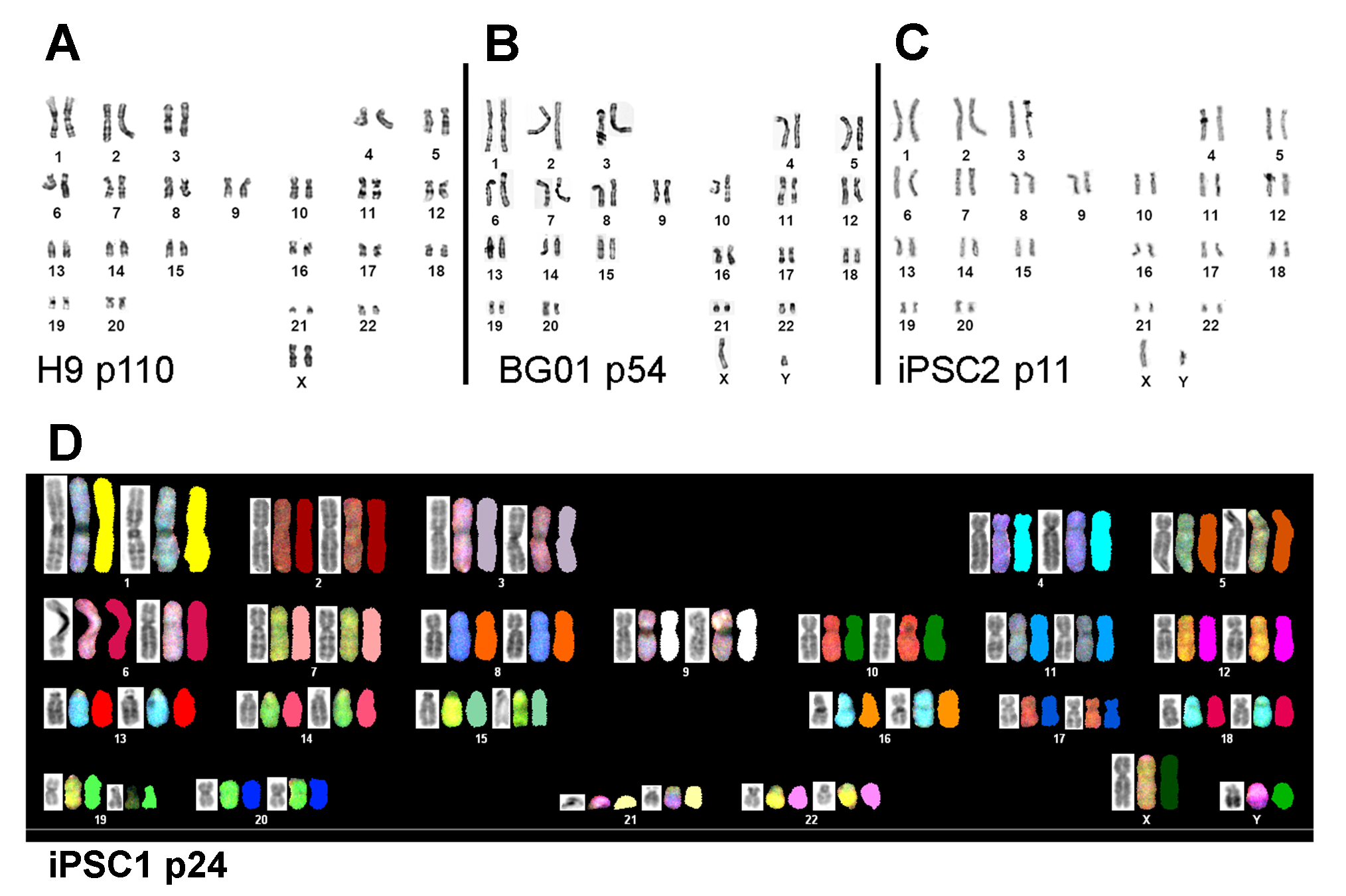

Supplement: Figure S2 — Karyotypes of investigated pluripotent cell lines. (A) H9 passage p 110, (B) BG01 p 54. BG01V (not shown) is a karyotypically abnormal (49, +12, +17 and XXY) long term cell culture variant originally isolated and characterized from BG01 cultures [2], and (C) iPSC2 p 11, as assessed by G-banding and (D) iPSC1 p 24, as assessed by spectral karyotyping (SKY) analysis. Both iPSC1 and iPSC2 were derived from human skin fibroblasts (CRL-2097) [3] or human lung fibroblasts (IMR90), respectively. The karyotypes examined for all these cells manifested 46 chromosomes in greater than 90% of the metaphase cells analyzed until at least p 110 for H9, p 54 for BG01, p 24 for iPSC1 and passage 11 for iPSC2. (TIF) [file pone.0030541.s002.tif]

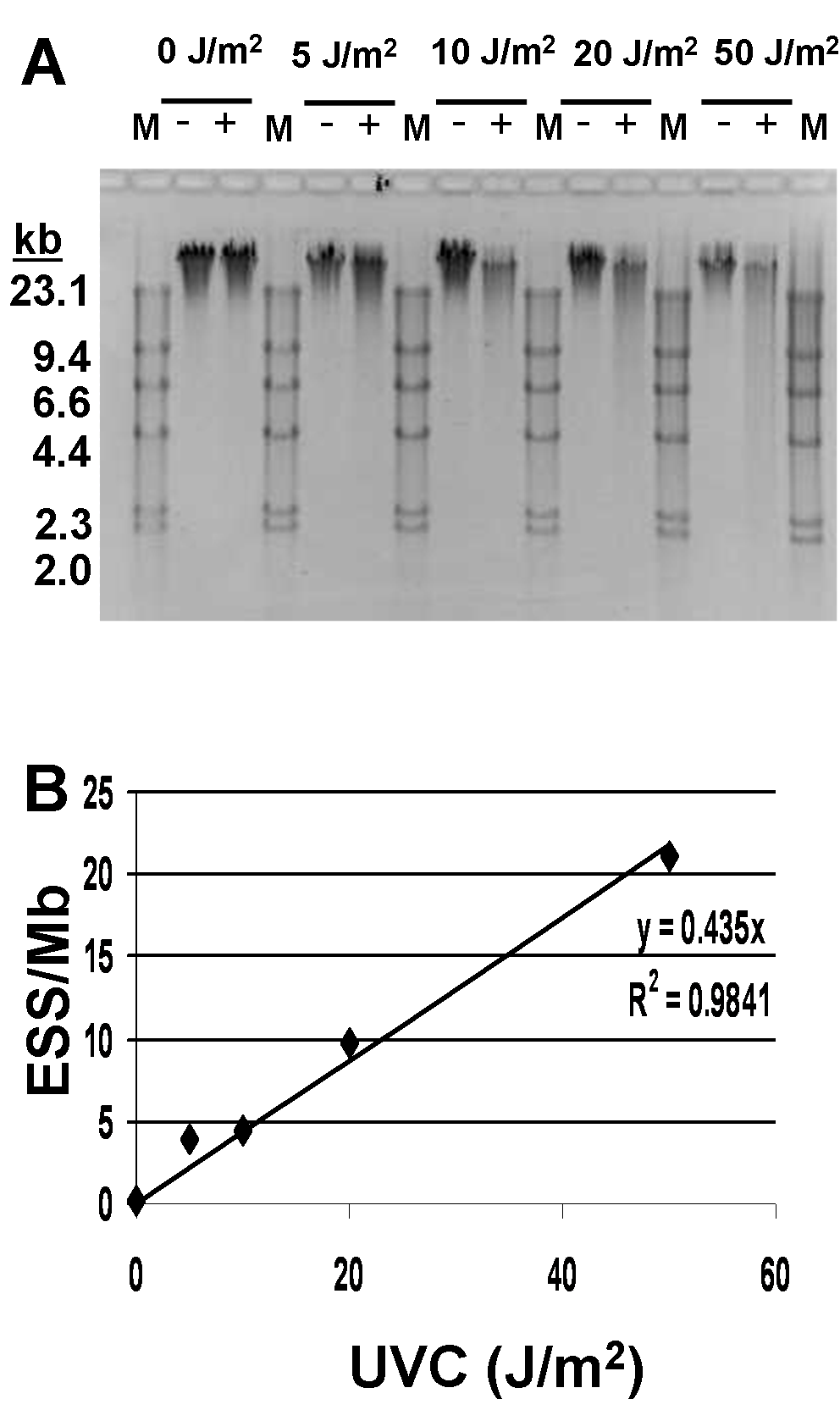

Supplement: Figure S3 — Analysis of CPD incidence in UVC-irradiated λ DNA. UVC-irradiated Bacteriophage λ DNA was subjected to alkaline gel analysis (A) and quantification (B) of UVC-induced enzyme sensitive sites per mega base (ESS/Mb) was conducted. Hind III-digested lamda DNA are used as DNA markers. (TIF) [file pone.0030541.s003.tif]

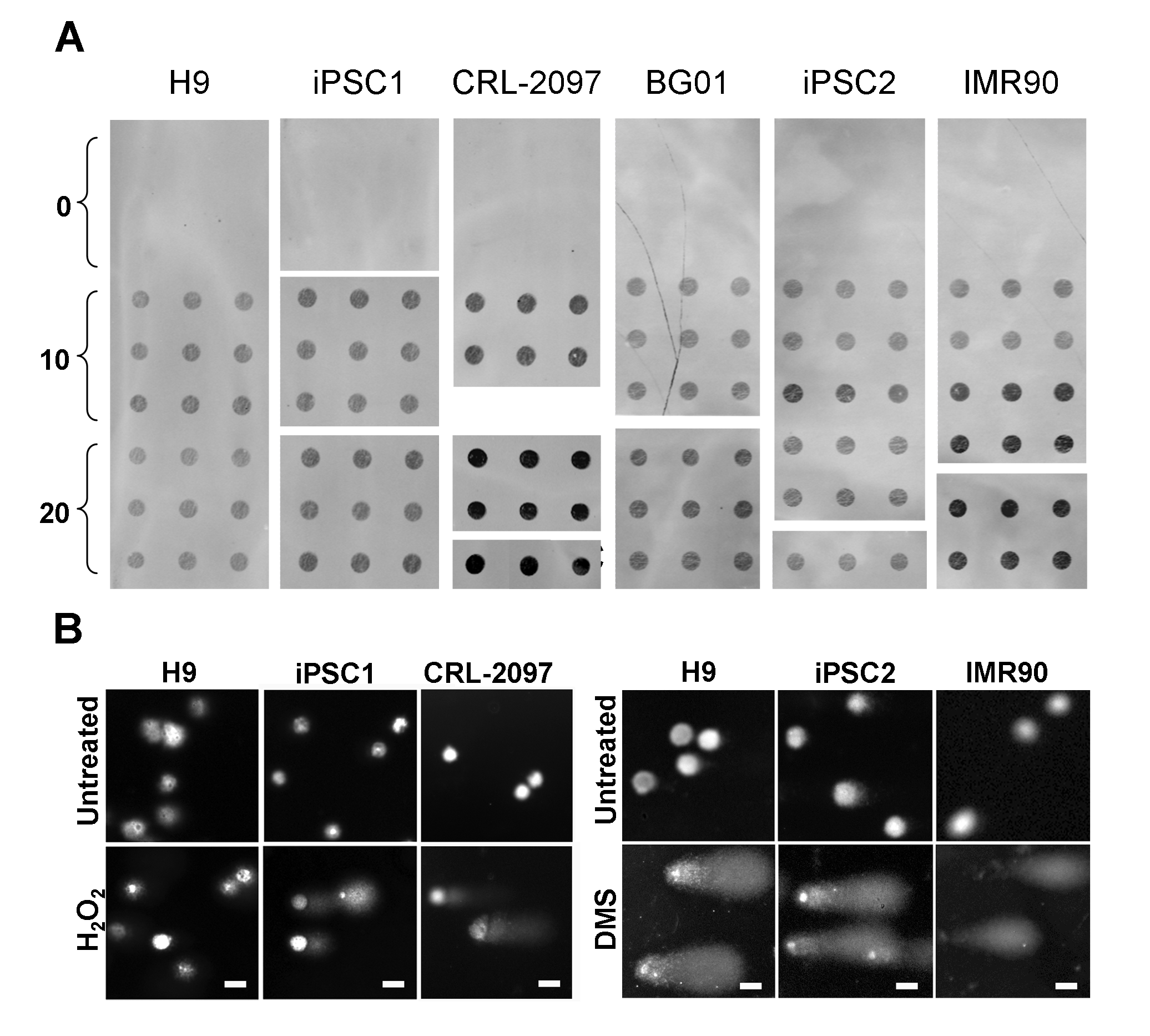

Supplement: Figure S4 — UVC, H2O2 or DMS-induced damage in hESC, iPSC and fibroblast cells. (A) Dot blot of UVC-induced (10 or 20 J/m2) CPD adducts in pluripotent cells and fibroblasts, quantified in TotalLab. (B) Comet assays of hESCs (H9), iPSCs (iPSC1), or human skin fibroblasts (CRL-2097) treated with H2O2 (100 µM). Untreated cells were used as controls. (C) Comet assays of hESCs (H9), iPSCs (iPSC2), or human skin fibroblasts (IMR90) treated with DMS (50 µM). (TIF) [file pone.0030541.s004.tif]

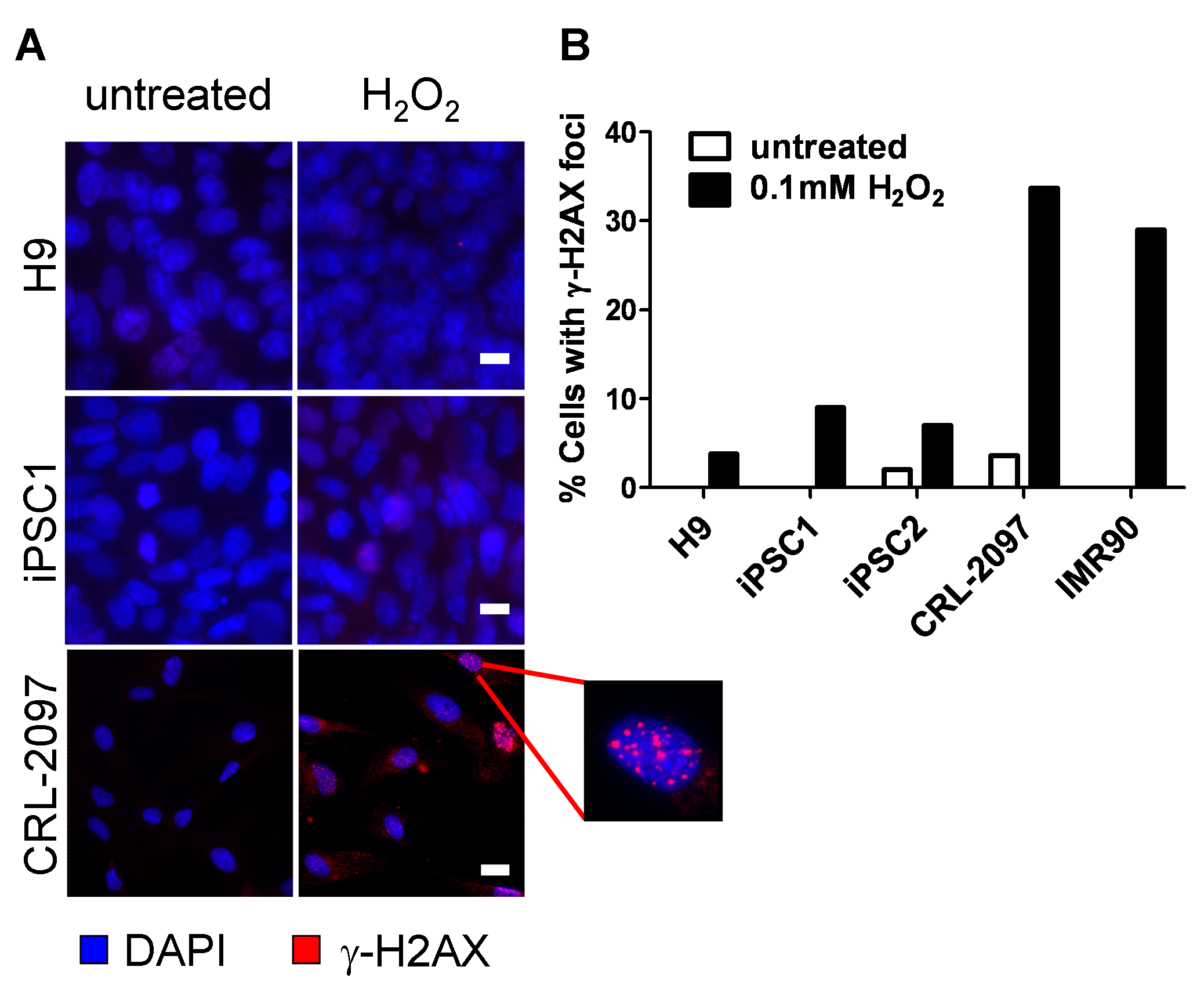

Supplement: Figure S5 — Evaluation of γH2AX foci formation in response to treatment with H2O2. (A) Fluorescence images of hESCs (H9), iPSCs (iPSC1) and fibroblasts (CRL-2097) stained for γH2AX foci after treatment with 100 µM H2O2 (4°C for 30 min). The expanded cell shows the foci as examined in the individual cells. Controls are untreated samples. Bars are 20 µm. (B) Quantification of percent of cells with greater than 4 γH2AX foci. (TIF) [file pone.0030541.s005.tif]

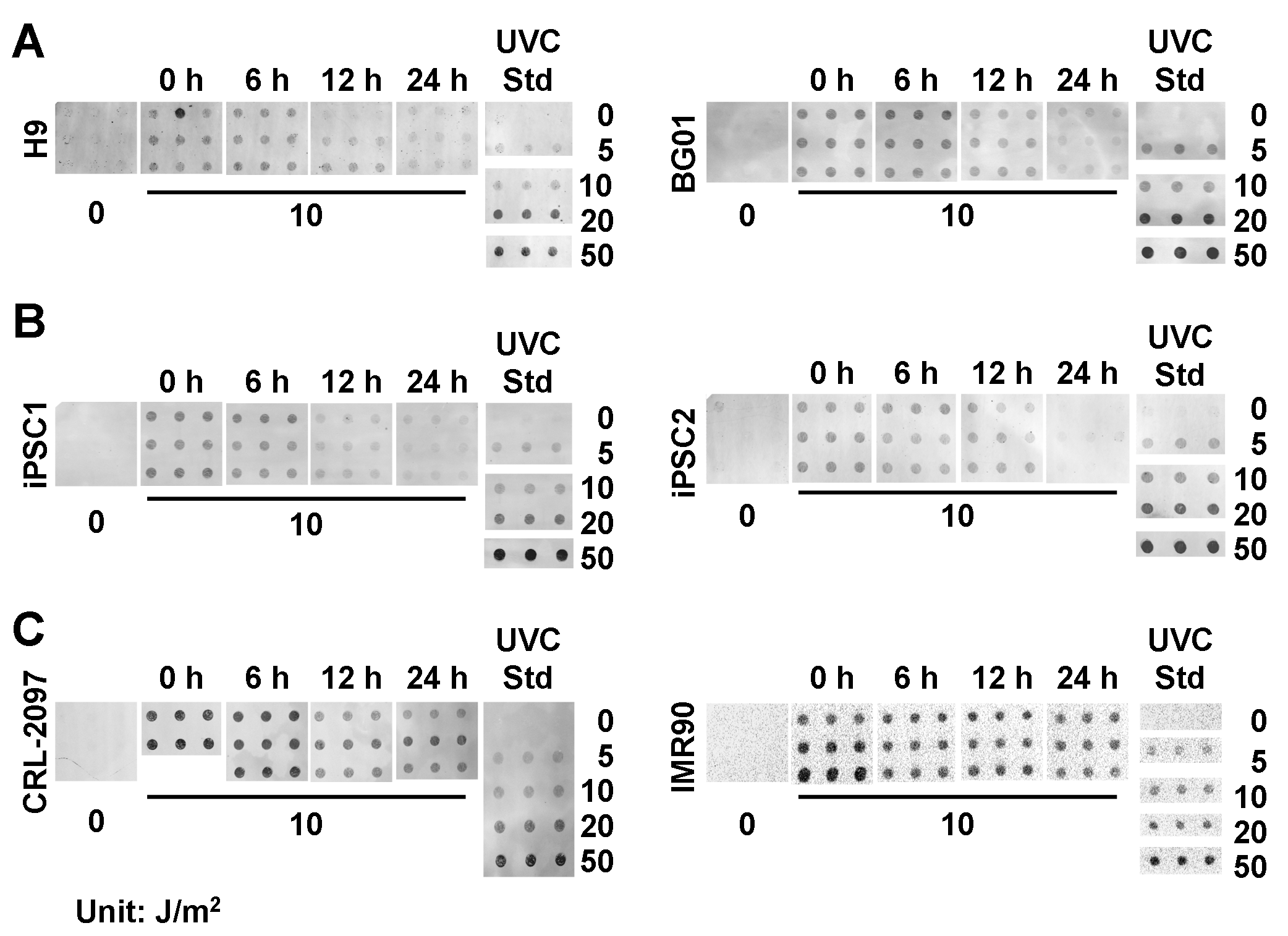

Supplement: Figure S6 — Dot blot assay data for global genome-nucleotide excision repair of UVC-induced cyclobutane pyrimidine dimers. Dot blot images of CPD repair time course in (A) hESC (H9 and BG01), (B) iPSC (iPSC1 and iPSC2) and (C) fibroblast (CRL-2097 and IMR90) cells following 10 J/m2 UVC treatment. Only adherent cells were used in the assay. Quantification of enzyme sensitive sites per mega base was determined using standards loaded on each individual blot. (TIF) [file pone.0030541.s006.tif]

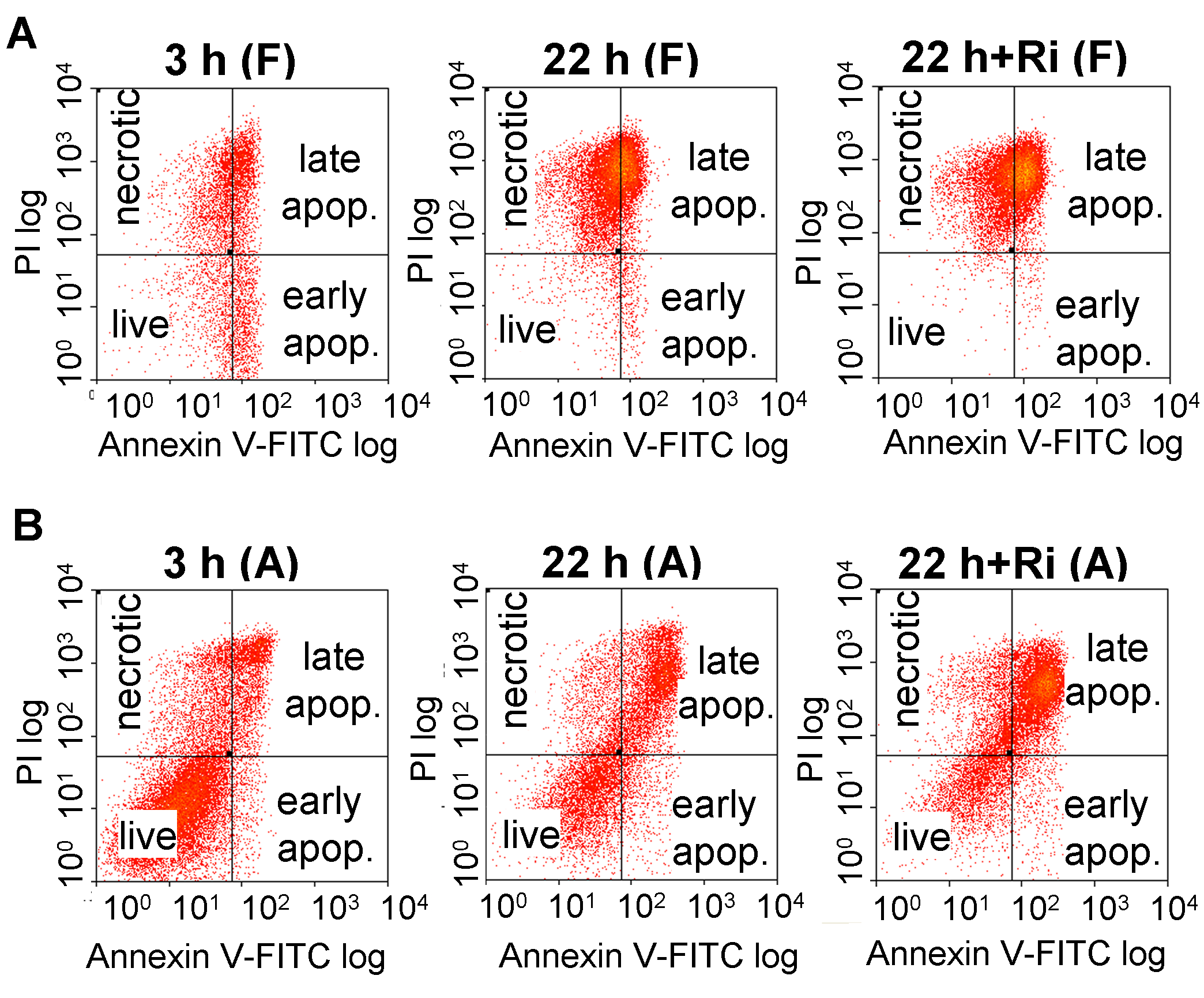

Supplement: Figure S7 — FACS analysis of the H9 cell states post UVC irradiation (10 J/m2). At the time points indicated, floating (F) and adherent (A) H9 cells were collected by centrifugation or accutase treatment followed by centrifugation and incubated with Annexin V-FITC and/or PI. Cells are divided by quadrants into live (FITC−, PI−), early apoptotic (FITC+, PI−), late apoptotic (FITC+, PI+) or necrotic (FITC−, PI+) sections. The quantification is shown in Figure 7B . (TIF) [file pone.0030541.s007.tif]

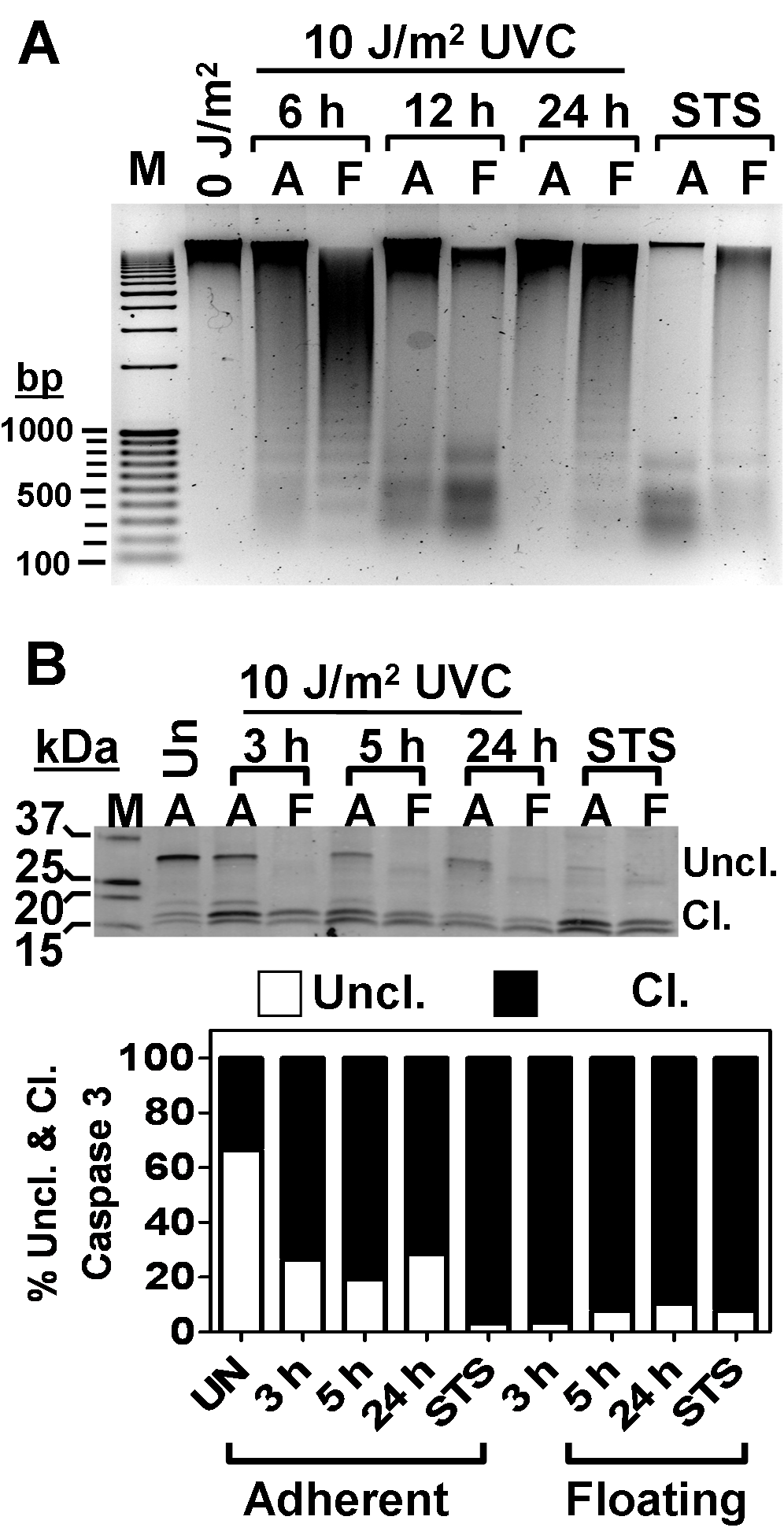

Supplement: Figure S8 — UVC-induced apoptosis in induced pluripotent stem cells. (A) DNA fragmentation analysis of UVC-irradiated iPSC2 cells. STS, staurosporine; S, supernatant; F, floating cells; A, adherent cells (B) Caspase 3 cleavage in adherent and floating cells. Upper panel: Western blot of caspase 3 cleavage in iPSC2 cells, treated with 10 J/m2 UVC (6, 12 and 24 h) or staurasporine (3 h), using near-infrared detection. Uncleaved (Uncl.); Cleaved (Cl.); Floating cells (F); Adherent cells (A). Note that there are no floating cells prior to treatment. Lower panel: analysis of Western blots comparing uncleaved (Uncl.) and cleaved (Cl.) bands for caspase 3. (TIF) [file pone.0030541.s008.tif]
