# Supplementary material for: DNA Repair in Human Pluripotent Stem Cells Is Distinct from That in Non-Pluripotent Human Cells
Source: PLoS One. 2012 Mar 6;7(3):e30541. doi: 10.1371/journal.pone.0030541 (PMC3295811; doi:10.1371/journal.pone.0030541)
Supplement: Table S1 — Microsatellite markers for MSI analysis. (DOC) [file pone.0030541.s009.doc]

**Table S1. Microsatellite markers for MSI analysis**

| **Name &**  **Size (bp)** | **Chromosome location** | **Repeating unit** | **^1^ Primer sequence 5’🡪3’**  **F: forward, R: reverse** | **Nearby located gene** |
| --- | --- | --- | --- | --- |
| ^2^BAT26  (~ 120) | 2p16 | Mononucleotide | F: TGACTACTTTTGACTTCAGCC  R: AACCATTCAACATTTTTAACCC | *hMSH2* |
| ^2^ D2S123  (197 ~ 220) | 2p16 | Dinucleotide | F: AAACAGGATGCCTGCCTTTA  R: GGACTTTCCACCTATGGGAC | *hMSH2* |
| ^2^D17S250  (151 ~ 169) | 17q11.2-17q12 | Dinucleotide | F: GGAAGAATCAAATAGACAAT  R: GCTGGCCATATATATATTTAAACC | *NF1* |
| ^2^D5S346  (96 ~ 122) | 5q21-5q22 | Dinucleotide | F:ACTCACTCTAGTGATAAATCGGG  R:AGCAGATAAGACAGTATTACTAGTT | *APC* |
| ^3^D3S1619  (~ 167) | 3p24.2-3p22 | Dinucleotide | F: GTCCTGCAAGACTCATTG  R: TTGCTAGGATGGTTGTTTTC | *hMLH1* |
