# Supplementary material for: DNA Repair in Human Pluripotent Stem Cells Is Distinct from That in Non-Pluripotent Human Cells
Source: PLoS One. 2012 Mar 6;7(3):e30541. doi: 10.1371/journal.pone.0030541 (PMC3295811; doi:10.1371/journal.pone.0030541)
Supplement: Table S2 — Summary of DNA repair rates/capacities of hPSCs and HFs in multiple DNA repair pathways investigated. The rates/capacities for all the lines are relative to the rates/capacities in IMR-90 fibroblasts (1.0). Values are mean ± Standard Deviation. Note that the repair rates are directly comparable down a column and not across rows. (DOC) [file pone.0030541.s010.doc]

**Table S2. Summary of DNA repair rates/ capacities of hPSCs and HFs in multiple DNA repair pathways investigated.**

| **Line** | **Designation** | **GG-NER^1^** | **TC-NER^2^** | **BER^2^** | **NHEJ^2^** | **SSA^2^** |
| --- | --- | --- | --- | --- | --- | --- |
| **H9** | **hESC** | 2.96±0.12 | 1.87±0.09 | 5.38±0.13 | 1.54±0.08 | 0.63±0.08 |
| **NSC09** | **NSC** |  |  |  | 1.16±0.27 | 0.48±0.04 |
| **BG01** | **hESC** | 2.66±0.28 | 1.48±0.08 | 1.22±0.01 | 0.91±0.13 | 0.48±0.02 |
| **BG01V** | **hESC** |  | 1.19±0.11 | 3.93±0.16 | 1.08±0.08 | 0.68±0.13 |
| **iPSC1** | **iPSC** | 3.30±0.14 | 0.47±0.01 | 0.46±0.01 | 2.21±0.44 | 0.45±0.03 |
| **iPSC2** | **iPSC** | 3.48±0.30 | 1.50±0.08 | 5.56±0.06 | 0.77±0.14 | 0.28±0.02 |
| **IMR90** | **Lung Fibroblasts** | 1.00±0.06 | 1.00±0.03 | 1.00±0.02 | 1.00±0.02 | 1.00±0.06 |
| **CRL-2097** | **Skin Fibroblasts** | 2.39±0.13 | 1.38±0.05 | 2.79±0.06 |  |  |
| **HF02** | **Skin Fibroblasts** |  | 1.25±0.12 | 2.13±0.26 | 1.29±0.28 | 0.97±0.10 |
