# Supplementary material for: DNA Repair in Human Pluripotent Stem Cells Is Distinct from That in Non-Pluripotent Human Cells
Source: PLoS One. 2012 Mar 6;7(3):e30541. doi: 10.1371/journal.pone.0030541 (PMC3295811; doi:10.1371/journal.pone.0030541)
Supplement: Materials and Methods S1 — (DOC) [file pone.0030541.s013.doc]

**Supplemental Material and Methods S1**

**Immunohistochemistry**

Antibodies were purchased from the following vendors: Santa Cruz: rabbit anti-Oct4; Millipore: mouse anti-Oct4, goat anti-SOX2, rabbit and mouse anti-γH2AX; Abcam: rabbit anti-NANOG, mouse anti-Dnmt3b; Developmental Studies Hybridoma Bank: mouse anti-SSEA4; and Invitrogen: Alexa 488, 568 and 647 donkey anti-mouse, rabbit and goat IgG(H+L). Cells were fixed (20 min, RT) in 4% paraformaldehyde in 1X PBS (150 mM NaCl, 20 mM sodium phosphate [pH 7.4]), permeabilized with 1X PBS, 0.1% Triton X-100 (2 min, RT) and incubated with 1% BSA/1X PBS (w/v) (1 h, RT). Incubation with primary antibodies (1–2 μg/ml final concentration) was in 1% BSA/1X PBS (overnight, 4°C) followed by washes in 1X PBS (2 x 5 min) and incubation with appropriate fluorescently-labeled secondary antibodies in 1% BSA/1X PBS (6 μg/ml, 45 min, RT). ProLong gold antifade reagent with DAPI (Invitrogen) was added to the cells before they were visualized. An Olympus IX81 automated inverted or Zeiss inverted LSM510 META 2-Photon confocal Microscope was used for imaging.

**Chromosome Preparation**

hESC cultures at 80% confluence were incubated (1 h, 37° C) with colchicine (2 μg/ml), washed with 1X PBS and dissociated with 0.25% Trypsin-EDTA, to produce a single cell suspension. Cells were quantified by hemocytometry, pelleted in a microfuge tube (960 x g) and resuspended in serum-free DMEM at 1 x 10^7^cells/mL. Samples were slowly diluted 1:6 with 0.075 M KCl hypotonic solution and incubated (20 min, RT), after which cold Carnoy’s fixative (3:1 methanol: glacial acetic acid) was added drop-wise with gentle mixing to 1.2 ml final volume. Cells were held in Carnoy’s fixative (1 h, RT or overnight, 4° C), centrifuged (1300 x g) and then resuspended in cold Carnoy’s fixative. This was repeated at least twice to achieve complete dehydration. Following final resuspension in 200 µL Carnoy’s fixative, cell suspensions were dropped onto clean slides and dried in a fume hood.

**Karyotyping**

Slides containing high quality metaphase spreads were aged for 2–6 days in the dark before G-Banding, which was produced by incubation for 5–25 s with 100 µL 0.05% Trypsin/EDTA under a coverslip. Trypsinization was quenched by dipping the slide in serum-free DMEM, thereby allowing the coverslip to fall away from the slide. Slides for various exposure times were stained with fresh 4% Giemsa Stain in Gurr’s buffer (25 min), rinsed twice in water, shaken, and dried at an angle. Slides were mounted with coverslips, and examined using bright field illumination with a Zeiss Upright Axioplan microscope and a 100X/1.30 oil immersion lens. Properly spread, unscattered metaphase spreads were counted and photographed. Karyograms were made according to the International System for Human Cytogenetic Nomenclature (ISCN 2005).

**Alkaline gel analysis**

Radiant incidence was measured and used to calculate the UVC exposure times to achieve the desired levels of irradiation for the various λ DNA standards. Approximately 1 μg irradiated DNA standards was incubated with 5 unit T4 Endonuclease V (Trevigen), 30 μL digest reaction (1M Tris-HCl [pH 7.5] and 100 mM EDTA), at 37° C for 1 h. After digestion, equal volumes of DNA standards (0.5–1 μg) were combined with Denaturing Solution (100 mM NaOH, 4 mM EDTA) and 2.5X Denaturing Gel Loading Buffer (1M NaOH, 50% Glycerol and 0.05% Bromocresol Green) and incubated at RT for 15–20 min. Samples were loaded on an alkaline gel (0.5 g agarose, 100 mL Alkaline Gel Solution [50 mM NaOH, 4 mM EDTA]) and run at 75 V for 2.5 h in Alkaline Electrophoresis Buffer (30 mM NaOH, 2 mM EDTA). Following electrophoresis, the alkaline gel was incubated at RT in 500 mL Alkaline Gel Neutralization Buffer (500 mM Tris-HCl [pH 8.0], 60.57 g Tris) and stained with 1 μg/mL ethidium bromide solution for at least 30 min to visualize DNA. The number of enzyme sensitive sites per mega base was determined for each standard.

**Supplemental References**

1. Chan EM, Ratanasirintrawoot S, Park IH, Manos PD, Loh YH, et al. (2009) Live cell imaging distinguishes bona fide human iPS cells from partially reprogrammed cells. Nat Biotechnol 27: 1033-1037.

2. Zeng X, Chen J, Liu Y, Luo Y, Schulz TC, et al. (2004) BG01V: a variant human embryonic stem cell line which exhibits rapid growth after passaging and reliable dopaminergic differentiation. Restor Neurol Neurosci 22: 421-428.

3. Yu J, Vodyanik MA, Smuga-Otto K, Antosiewicz-Bourget J, Frane JL, et al. (2007) Induced pluripotent stem cell lines derived from human somatic cells. Science 318: 1917-1920.
